# A general theory of far-field optical microscopy image formation and resolution limit using double-sided Feynman diagrams

**DOI:** 10.1038/s41598-020-73584-1

**Published:** 2020-10-19

**Authors:** Naoki Fukutake

**Affiliations:** Nikon Corporation, 471 Nagaodai-cho, Sakae-ku, Yokohama-city, Kanagawa 244-8533 Japan

**Keywords:** Optics and photonics, Physics

## Abstract

Optical resolution of far-field optical microscopy is limited by the diffraction of light, while diverse light-matter interactions are used to push the limit. The image resolution limit depends on the type of optical microscopy; however, the current theoretical frameworks provide oversimplified pictures of image formation and resolution that only address individual types of microscopy and light-matter interactions. To compare the fundamental optical resolutions of all types of microscopy and to codify a unified image-formation theory, a new method that describes the influence of light-matter interactions on the resolution limit is required. Here, we develop an intuitive technique using double-sided Feynman diagrams that depict light-matter interactions to provide a bird’s-eye view of microscopy classification. This diagrammatic methodology also allows for the optical resolution calculation of all types of microscopy. We show a guidepost for understanding the potential resolution and limitation of all optical microscopy. This principle opens the door to study unexplored theoretical questions and lead to new applications.

## Introduction

Ernst Abbe established the image formation theory in optical microscopy and derived the well-known optical resolution formula, d = λ/2NA. This resolution limit corresponds to a frequency cut-off of 2NA/λ, where λ is the wavelength of light and NA is the numerical aperture of the microscope objective^1^. Abbe’s theorem is obeyed by classical microscopy techniques such as bright-field microscopy^2^, phase contrast microscopy^3,4^, differential interference microscopy^5^ and dark field microscopy^6^, as well as by newer microscopy techniques such as relief contrast microscopy^7^, digital holographic microscopy^8^, and optical coherence tomography (OCT)^9^. These microscopy use only χ^(1)^-derived light-matter interactions, such as linear absorption, transmission, and reflection, where χ^(1)^ is the electrical susceptibility. Considering three-dimensional (3-D) optical resolution in transmission microscopy that uses χ^(1)^-derived interactions, it is known that the missing cone problem exists in the spatial-frequency domain^10^. The missing cone expresses the frequency region that cannot be resolved, which causes poor optical resolution in the *z* (optical-axis) direction. Although Abbe’s definition of resolution limit is still used as the standard, it is not well known that the frequency limit 2NA/λ applies only to microscopy techniques that utilize χ^(1)^-derived interactions.

Recently, many types of optical microscopy have been developed that use a variety of light-matter interactions, such as two-photon excited fluorescence^11^, second-order harmonic generation^12^, third-order harmonic generation^13^, coherent anti-Stokes Raman scattering^14^, stimulated Raman scattering^15^, and so on. When nonlinear susceptibility χ^(*i*)^ are included, the resolution limit may surpass 2NA/λ and the missing cone problem can be overcome, where *i*≧2 for higher-order light-matter interactions^16^. This implies that the higher-order interactions, even linear fluorescence, which is an χ^(3)^-derived interaction, cannot be addressed using Abbe’s formula. Indeed, fluorescence confocal microscopy possesses a frequency cutoff of 4NA/λ^10^, presuming that the wavelengths of the excitation beam and fluorescence are equal. The image formation theory of fluorescence microscopy exists, but it is formulated by using different approach. While the Abbe’s formula is based on the diffraction of light, the fluorescence microscopy theory does not apply the concept of diffraction because fluorescence appears to be unrelated to the diffraction. However, we can expand the concept of diffraction to even fluorescence by including the vacuum field in the light-matter interaction. Nevertheless, we encounter the implication of incorrectly applying Abbe’s formula to χ^(*i*)^-derived interactions (*i*≧2). A typical example of inappropriate interpretation is that the frequency cutoff of 2NA/λ of wide-field fluorescence microscopy originates from the diffraction. This frequency cutoff happens to become equal to that of bright-field microscopy, which does originate from the diffraction. Thus, we need to expand the diffraction theory in χ^(1)^-derived interactions to also the high-order interactions. Until now, the image formation theories do not exist that attempt to generalize the Abbe’s formula into a more unified theory of light-matter interactions, except for our previous works^16–18^, which address only laser microscopy. Our new theory covers both the Kohler illumination systems and laser excitation systems.

In this study, we formulate a new universally applicable theory, and derive the rules for calculating the resolution limit for all optical microscopy techniques that use arbitrary light-matter interactions. To address all light-matter interactions in the same framework, we use double-sided Feynman diagrams that describe how the density matrix of molecular ensemble time-evolves as interacting with lights (electric fields)^19^. Under our theory, the resolution limit can be calculated using the diagram. In nonlinear optics, the Feynman diagram method was originally developed for classifying light-matter interactions and calculating the amplitude and phase of χ^(*i*)^^20^. We extend the applicability of the diagram method in order to calculate the resolution limit. All linear, nonlinear, coherent, and incoherent light-matter interactions can be described by a diagram, which includes some arrows representing electric fields, as introduced in Ref. 19. We show that each arrow corresponds to the 3-D pupil function, following the rule we derived (Table 1 in “Results”). We also define the 3-D aperture as the best indicator of the resolution limit. Our major finding is that the 3-D aperture can be derived from the convolution of all of the corresponding 3-D pupil functions in the diagram of interest. In some cases, the 3-D pupil function is permitted to apply an apodization technique (pupil engineering), and in other cases, plural-order interactions occurring simultaneously are considered. According to our theory, if no a priori information on the χ^(*i*)^ distribution in the sample exists, the type of light-matter interaction determines the resolution limit of far-field optical microscopy. While even individual precise image formation theory of the microscopy that uses a certain light-matter interaction does not exists except for χ^(1)^-derived interaction and fluorescence, we unify all interactions into one framework for microscopy image formation. Our novel general theory includes Abbe’s formula as a special case for the lowest order (χ^(1)^) interactions, and addresses all optical microscopy that uses arbitrary high-order interactions. To achieve the universally applicable theory, we reframe the diffraction as the lowest order quasi-phase-matching (QPM)^21^, and include the vacuum field as one of the excitation fields responsible for the light-matter interaction.

**Table 1:**
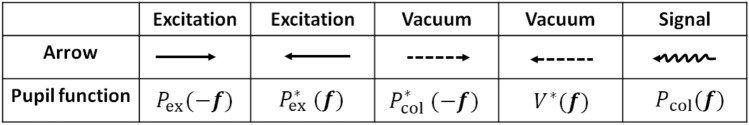
Correspondence rule between arrows and 3-D pupil functions in confocal microscopy.

$P_{{{\text{ex}}}} \left( {\varvec{f}} \right)$, $P_{{{\text{col}}}} \left( {\varvec{f}} \right)$, and $V\left( {\varvec{f}} \right)$ are the 3-D pupil functions for the excitation system, signal-collection systems, and the vacuum field, respectively (Only arrows in relevant Feynman diagram are convolved to find the 3-D aperture, irrespective of the presence of the LO).

## Results

### Generalization of Abbe’s image formation theory

To generalize Abbe’s image formation theory into a unified theory that can address all light-matter interactions, we expand some concepts as follows: (i) a target to be observed has *i*-th order linear/nonlinear susceptibility ${\upchi }^{\left( i \right)}$, (ii) diffraction is replaced by QPM, and (iii) the vacuum field is included as one of the excitation fields. Considering that the QPM is a broader concept than the diffraction, we can exactly regard the diffraction as the lowest order QPM. Although it is not well known that the vacuum field acts as one of the excitation field in incoherent light-matter interactions, we include the vacuum field in our framework. For simplicity, the first-order Born approximation is applied. In this approximation, both multiple scattering and depletion of the beam are neglected as this usually holds true for nearly transparent samples, such as biological specimens^22,23^. If multiple scattering and depletion occur in non-transparent samples, then the acquired image will be blurred to some extent, causing the decrease in signal-to-noise ratio. We assume that both the excitation and signal-collection systems are 1 × magnification systems, which does not change the essence of the image-forming properties. To focus on the resolution limit, our model employs the scalar diffraction theory^22^ that neglects the influence of polarized light, which does not affect the frequency cutoff.

We define an object as a complex susceptibility distribution ${\upchi }^{\left( i \right)} \left( {\varvec{x}} \right)$, where ***x*** = (*x*, *y*, *z*) is a coordinate in the object space, and *i* is the order of light-matter interaction. This order coincides with the number of excitation fields in a double-sided Feynman diagram, as discussed in the next section. We also expand the concept of diffraction into QPM, defining the ${\upchi }^{\left( 1 \right)}$-diffraction as the lowest order. In QPM, the object is assumed to contain a 3-D cosine-shaped ${\upchi }^{\left( i \right)}$ grating, $\cos 2\pi {\varvec{f}}_{{\text{g}}} \cdot {\varvec{x}} = 1/2\left\{ {e^{{i2\pi {\varvec{f}}_{{\text{g}}} \cdot {\varvec{x}}}} + e^{{ - i2\pi {\varvec{f}}_{{\text{g}}} \cdot {\varvec{x}}}} } \right\}$, where ${\varvec{f}}_{{\text{g}}}$ is a grating vector, as shown in Supplementary Figure 3a. The ${\upchi }^{\left( i \right)} \left( {\varvec{x}} \right)$ distribution in the object is composed of many gratings with different grating vectors and usually takes the form of either a purely real or purely imaginary function^20,21^. For convenience, hereafter we will discuss image formation by considering the Fourier components of ${\upchi }^{\left( i \right)} \left( {\varvec{x}} \right)$, i.e., $e^{i\theta } e^{{i2\pi {\varvec{f}}_{{\text{g}}} \cdot {\varvec{x}}}}$, with an arbitrary phase, $\theta$.

We begin with Abbe’s theory, which was formulated for bright-field microscopy with the Kohler illumination system^2^. This formulation is also applicable for phase contrast microscopy^3,4^, differential interference contrast microscopy^5^, and dark-field microscopy^6^. Two-dimensional (2-D) detectors, such as a charge-coupled device (CCD) or complementary metal–oxide–semiconductor (CMOS), are often employed in place of eyepiece. As a result, three-dimensional (3-D) images can be obtained by scanning a sample stage in the optical axis direction. In optical microscopy with the Kohler illumination system and a 2-D detector, the optical resolution is determined by the illumination NA and the pixel size of the 2-D detector, without considering the objective NA or light wavelength. For example, the image intensity of bright-field microscopy ${\text{I}}_{{\text{K}}} \left( {\varvec{x}^{\prime}} \right)$ is given as follows^22^:

1 $$ {\text{I}}_{{\text{K}}} \left( {{\varvec{x}}^{\prime } } \right) = \int {\left| {\int {P_{{{\text{ill}}}} \left( {\varvec{f}} \right)e^{{i2\pi {\varvec{f}} \cdot {\varvec{x}}}} \chi^{\left( 1 \right)} \left( {\varvec{x}} \right)h_{{{\text{col}}}} \left( {{\varvec{x}}^{\prime } - {\varvec{x}}} \right)d^{3} {\varvec{x}}} } \right|^{2} } d^{3} {\varvec{f}}, $$

where $h_{{{\text{col}}}} \left( {\varvec{x}^{\prime} - {\varvec{x}}} \right)$ is an amplitude spread function (ASF) of the image-formation (signal-collection) system, $P_{{{\text{ill}}}} \left( {\varvec{f}} \right)$ represents a 3-D pupil function of the Kohler illumination system, and ${\varvec{f}}$ is a spatial frequency. $P_{{{\text{ill}}}} \left( {\varvec{f}} \right)$ is assumed to have a uniform modulus but actually has a random phase at each frequency ${\varvec{f}}$ because of the nature of an incoherent surface light source. This means that the sample is illuminated by countless plane waves with different incident angles as described by $P_{{{\text{ill}}}} \left( {\varvec{f}} \right)e^{{i2\pi {\varvec{f}} \cdot {\varvec{x}}}}$ that do not interfere with one another (Supplementary Figure 3b). By defining $P_{{{\text{ill}}}} \left( {\varvec{f}} \right)$ in the spatial-frequency domain, this restricts the NA of the illumination system, $NA_{{{\text{ill}}}}$. Here, we assume that the pixel size of the 2-D detector is sufficiently small and that the electric permittivity is $\upvarepsilon _{0} = 1$.

Next, we consider laser excitation microscopy. For comparison, we assume the same physical quantity $\chi^{\left( 1 \right)} \left( {\varvec{x}} \right)$. Laser microscopy is composed of an excitation system that focuses the laser beam onto a sample and a signal-collection system that gathers the scattered light generated from the sample. A schematic of laser microscopy with its coordinate system is shown in Supplementary Figure 3c. In the following, we assume 3-D sample-stage scanning rather than laser scanning; however, this assumption does not influence the optical resolution. The signals are acquired point by point with a photodetector to reconstruct the 3-D image. The image intensity ${\text{I}}\left( {\varvec{x}^{\prime}} \right)$ at the sample-stage displacement ${\varvec{x}}^{\prime } = \left( {x^{\prime } ,y^{\prime } ,z^{\prime } } \right)$ is given by^17,18^:

2 $$ {\text{I}}\left( {\varvec{x}^{\prime}} \right) = \int \left| {\int h_{{{\text{ex}}}} \left( {\varvec{x}} \right)\chi^{\left( 1 \right)} \left( {{\varvec{x}} - \varvec{x}^{\prime}} \right)h_{{{\text{col}}}} \left( {{\varvec{x}}_{{\text{d}}} - {\varvec{x}}} \right)d^{3} {\varvec{x}}} \right|^{2} D\left( {{\varvec{x}}_{{\text{d}}} } \right)d^{3} {\varvec{x}}_{{\text{d}}} , $$

where $h_{{{\text{ex}}}} \left( {\varvec{x}} \right)$ is an ASF representing the electric field distribution in the sample formed by an excitation system, ***x***_d_ = (*x*_d_, *y*_d_, *z*_d_) is the detection position, and $D\left( {{\varvec{x}}_{{\text{d}}} } \right)$ represents a 3-D function describing the detector size that has a delta-function characteristic in the *z* direction.

### Unified image formation formulas

To generalize all forms of optical microscopy, which includes Kohler illumination and linear/nonlinear laser excitation microscopy, into one expression, we deal with all microscopy in the same manner. First, we rewrite the formula for Kohler illumination microscopy Eq. (1) as shown below:

3 $$ \begin{aligned} {\text{I}}_{{\text{K}}} \left( { - \varvec{x}^{\prime}} \right) & = \int \left| {\int P_{{{\text{ill}}}} \left( {\varvec{f}} \right)e^{{i2\pi {\varvec{f}} \cdot {\varvec{x}}}} \chi^{\left( 1 \right)} \left( {{\varvec{x}} + {\varvec{x}}^{\varvec{^{\prime}}} } \right)h_{{{\text{col}}}} \left( { - {\varvec{x}}} \right)d^{3} {\varvec{x}}} \right|^{2} d^{3} {\varvec{f}} \\ & = \left| {\int K\left( {\varvec{x}} \right)\chi^{\left( 1 \right)} \left( {{\varvec{x}} + {\varvec{x}}^{\varvec{^{\prime}}} } \right)h_{{{\text{col}}}} \left( { - {\varvec{x}}} \right)d^{3} {\varvec{x}}} \right|^{2} , \\ \end{aligned} $$

where $K\left( {\varvec{x}} \right)$ represents an excitation electric field and is a Fourier transform of $P_{{{\text{ill}}}} \left( {\varvec{f}} \right)$, which has the characteristic of a random phase, i.e., $P_{{{\text{ill}}}} \left( {{\varvec{f}}_{1} } \right)P_{{{\text{ill}}}}^{*} \left( {{\varvec{f}}_{2} } \right) = \delta \left( {{\varvec{f}}_{1} - {\varvec{f}}_{2} } \right)\left| {P_{{{\text{ill}}}} \left( {{\varvec{f}}_{1} } \right)} \right|^{2}$. Note that $K\left( {{\varvec{x}}_{1} } \right)K^{*} \left( {{\varvec{x}}_{2} } \right)$ corresponds to the mutual intensity ${\Gamma }_{12} \left( {{\varvec{x}}_{1} - {\varvec{x}}_{2} } \right)$ ^22^. This equation can be written by using the following operators (see “Methods” for quantum-optical notation):

4 $$ {\text{I}}_{{\text{K}}} \left( { - \varvec{x}^{\prime} } \right) = \left\langle 0 \right|\left\langle {{\text{Cha}}} \right| \vdots \left| {\int \chi ^{{\left( 1 \right)}} \left( {\varvec{x} + \varvec{x}^{\prime} } \right)\hat{K}\left( \varvec{x} \right)\hat{a}_{{{\text{col}}}} \left( 0 \right)\hat{a}_{{{\text{sig}}}}^{ + } \left( \varvec{x} \right)d^{3} \varvec{x}} \right|^{2}  \vdots \left| {{\text{Cha}}} \right\rangle \left| 0 \right\rangle . $$

Here, $\left| 0 \right\rangle$ denotes the vacuum state, and the relation $\left\langle {{\text{Cha}}} \right|\hat{a}_{{{\text{ill}}}} \left( \varvec{f} \right)\left| {{\text{Cha}}} \right\rangle  = P_{{{\text{ill}}}} \left( \varvec{f} \right)$ (see Supplementary Note 8), i.e., $\left\langle {{\text{Cha}}} \right|\hat{K}\left( \varvec{x} \right)\left| {{\text{Cha}}} \right\rangle  = K\left( \varvec{x} \right)$, connects Eqs. (3) and (4), where $\left| {{\text{Cha}}} \right\rangle$ represents a chaotic state describing the incoherent surface light source. In Eq. (4), we define **0** as ${\varvec{x}}_{{\text{d}}} = \left( {0, 0, 0} \right)$. If we substitute $D\left( {{\varvec{x}}_{{\text{d}}} } \right) = \delta \left( {{\varvec{x}}_{{\text{d}}} } \right)$ in Kohler illumination microscopy, the image intensity obtained by any type of optical microscopy, including Kohler illumination and linear/nonlinear laser excitation microscopy, can be described as follows (see “Methods”):

5 $$ I\left( { - \varvec{x}^{\prime}} \right) = \left\langle {{\text{ex}}} \right| \vdots \int \left| { - i\hat{a}_{{{\text{lo}}}} \left( {{\varvec{x}}_{{\text{d}}} } \right) + \int \chi^{\left( i \right)} \left( {{\varvec{x}} + \varvec{x}^{\prime}} \right)\hat{E}_{{{\text{int}}}} \left( {\varvec{x}} \right)\hat{a}_{{{\text{col}}}} \left( {{\varvec{x}}_{{\text{d}}} } \right)d^{3} {\varvec{x}} } \right|^{2} D\left( {{\varvec{x}}_{{\text{d}}} } \right)d^{3} {\varvec{x}}_{{\text{d}}} \vdots \left | {{\text{ex}}} \right \rangle, $$

where $\left| {{\text{ex}}} \right\rangle$ represents an excitation condition that includes the vacuum field around the sample, and the operator for the local oscillator (LO), which is more intense than the signal field, $\hat{a}_{{{\text{lo}}}} \left( {{\varvec{x}}_{{\text{d}}} } \right)$ is introduced, with a Gouy phase shift^24^ of -*i*. The Gouy phase shift is a ${\uppi }/2$ phase difference between the LO and the signal emitted from a molecule, which arises during long-distance travel after leaving the focal point. The sign before $\varvec{x}^{\prime}$ in $I\left( { - \varvec{x}^{\prime}} \right)$ depends on the coordinate system in the image but does not affect the overall expression. In Kohler illumination microscopy, if the non-diffracted light (i.e. the illumination light itself) is much more intense than the diffracted light, the LO is present, corresponding to the first-order Born approximation. In “Results”, for reference, we also consider Kohler illumination microscopy in which the LO is absent as a partially coherent system. The microscope system determines whether the term corresponding to the LO vanishes or remains. If it vanishes, this can automatically be derived by calculating the expected value of the vacuum field operator. In the presence of LO, Eq. (5) leads to:

6 $$ I\left( { - {\varvec{x}}^{\prime}} \right) = Const. + i\int \chi^{\left( i \right)} \left( {{\varvec{x}} + \varvec{x}^{\prime}} \right)h_{{\text{t}}} \left( { - {\varvec{x}}} \right)d^{3} {\varvec{x}} + c.c., $$

and in the absence of LO, it leads to:

7 $$ {\text{I}}\left( { - \varvec{x}^{\prime}} \right) = \left| {\int \chi^{\left( i \right)} \left( {{\varvec{x}} + \varvec{x}^{\prime}} \right)h_{{\text{t}}} ^{\prime}\left( { - {\varvec{x}}} \right)d^{3} {\varvec{x}}} \right|^{2} , $$

where $h_{{\text{t}}} \left( { - {\varvec{x}}} \right) = \int \left\langle {{\text{ex}}} \right| \vdots \hat{E}_{{{\text{int}}}} \left( {\varvec{x}} \right)\hat{a}_{{{\text{lo}}}}^{ + } \left( {{\varvec{x}}_{{\text{d}}} } \right)\hat{a}_{{{\text{col}}}} \left( {{\varvec{x}}_{{\text{d}}} } \right) \vdots \left |{{\text{ex}}} \right \rangle D\left( {{\varvec{x}}_{{\text{d}}} } \right)d^{3} {\varvec{x}}_{{\text{d}}}$ and $h_{{\text{t}}} ^{\prime}\left( { - {\varvec{x}}} \right) = \int \left\langle {{\text{ex}}} \right| \vdots \hat{E}_{{{\text{int}}}} \left( {\varvec{x}} \right)\hat{a}_{{{\text{col}}}} \left( {{\varvec{x}}_{{\text{d}}} } \right) \vdots \left| {{\text{ex}}} \right\rangle D_{R} \left( {{\varvec{x}}_{{\text{d}}} } \right)d^{3} {\varvec{x}}_{{\text{d}}}$. Here, we define a detector function, $D_{{\text{R}}} \left( {{\varvec{x}}_{{\text{d}}} } \right)$, as $\left| {D_{{\text{R}}} \left( {{\varvec{x}}_{{\text{d}}} } \right)} \right|^{2} = D\left( {{\varvec{x}}_{{\text{d}}} } \right)$, where $D_{{\text{R}}} \left( {{\varvec{x}}_{{\text{d}}} } \right)$ has a random phase distribution. In either case, $h_{{\text{t}}} \left( {\varvec{x}} \right)$ or $h_{{\text{t}}} ^{\prime}\left( {\varvec{x}} \right)$ acts as the Green’s function of the total microscope system that generates the image (complex amplitude) from the object $\chi^{\left( i \right)} \left( {\varvec{x}} \right)$, and becomes the key function that determines the resolution limit.

### 3-D aperture for defining the resolution limit

In order to consider the optical resolution of all types of microscopy, we define a 3-D aperture $A\left( {\varvec{f}} \right)$ as the Fourier transform of $h_{{\text{t}}} \left( {\varvec{x}} \right)$ or $h_{{\text{t}}} ^{\prime}\left( {\varvec{x}} \right)$. The physical significance of the 3-D aperture is that it controls the rate of the Fourier components of $\chi^{\left( i \right)} \left( {\varvec{x}} \right)$ acquired through the microscope system. Information outside the 3-D aperture cannot reach the detector and cannot contribute to the image formation. The 3-D aperture is a concept similar to the optical transfer function (OTF)^25^, but it can always be defined even when the OTF of the microscopy of interest cannot be defined. For the purpose of comparing the optical resolution of all types of microscopy, the OTF cannot be used. Therefore, the 3-D aperture is a more fundamental physical quantity than the OTF that describes the optical resolution. Although the transmission cross coefficient (TCC) has been used to express the image-formation properties of partially coherent systems, such as bright-field microscopy^22^, the 3-D aperture is more helpful to intuitively understand partially coherent systems than the TCC. Even though the TCC can also be defined mathematically for any types of microscopy, it is difficult and less-helpful for the physical understanding of the resolution limit. Because the 3-D aperture is defined more straightforwardly than the TCC such that information cannot be acquired outside the frequency cutoff, it is the best indicator for the resolution limit.

We first consider microscopy in the presence of the LO. The Fourier transform of Eq. (6) is written as follows:

8 $$ \tilde{I}\left( {\varvec{f}} \right) = const.\delta \left( {\varvec{f}} \right) + i\widetilde{{\chi^{\left( i \right)} }}\left( {\varvec{f}} \right)A\left( {\varvec{f}} \right) - i\left\{ {\widetilde{{\chi^{\left( i \right)} }}\left( { - {\varvec{f}}} \right)} \right\}^{*} \left\{ {A\left( { - {\varvec{f}}} \right)} \right\}^{*} , $$

where $\widetilde{{\chi^{\left( i \right)} }}\left( {\varvec{f}} \right)$ is a Fourier transform of $\chi^{\left( i \right)} \left( {\varvec{x}} \right)$. The 3-D aperture is derived from the second term of Eq. (8); in this case, the third term is merely a Hermitian conjugate of the second term, which means that the third term does not contain additional information. In the presence of the LO, the OTF can always be defined by substituting in the Fourier transforms of the real and imaginary parts of $\chi^{\left( i \right)} \left( {\varvec{x}} \right)$. In other words, $\chi^{\left( i \right)} \left( {\varvec{x}} \right) = \chi_{{\text{r}}}^{\left( i \right)} \left( {\varvec{x}} \right) + i\chi_{{\text{i}}}^{\left( i \right)} \left( {\varvec{x}} \right)$ and $\widetilde{{\chi^{\left( i \right)} }}\left( {\varvec{f}} \right) = \widetilde{{\chi_{{\text{r}}}^{\left( i \right)} }}\left( {\varvec{f}} \right) + i\widetilde{{\chi_{{\text{i}}}^{\left( i \right)} }}\left( {\varvec{f}} \right)$ into Eq. (8) leads to:

9 $$ \tilde{I}\left( {\varvec{f}} \right) = const.\delta \left( {\varvec{f}} \right) + \widetilde{{\chi_{{\text{r}}}^{\left( i \right)} }}\left( {\varvec{f}} \right)OTF_{{\text{r}}} \left( {\varvec{f}} \right) + \widetilde{{\chi_{{\text{i}}}^{\left( i \right)} }}\left( {\varvec{f}} \right)OTF_{{\text{i}}} \left( {\varvec{f}} \right), $$

where it is used that $\widetilde{{\chi_{{\text{r}}}^{\left( i \right)} }}\left( {\varvec{f}} \right)$ and $\widetilde{{\chi_{{\text{i}}}^{\left( i \right)} }}\left( {\varvec{f}} \right)$ are Hermitian functions, and $OTF_{{\text{r}}} \left( {\varvec{f}} \right) = iA\left( {{ }\varvec{f}} \right){ } - iA^{*} \left( { - \varvec{f }} \right)$ and $OTF_{{\text{i}}} \left( {\varvec{f}} \right) = - A\left( {\varvec{f}} \right) - A^{*} \left( { - \varvec{f } } \right)$ represent the OTFs for the real and imaginary parts of $\chi^{\left( i \right)} \left( {\varvec{x}} \right)$, respectively. Thus, two types of OTFs are defined, which holds true in microscopy with the LO. Although χ^(*i*)^(***x***) is generally a complex function, in most cases χ^(*i*)^(***x***) is either a pure real or pure imaginary function. In this case, *OTF*_r_ (***f***) and *OTF*_i_ (***f***) become well-defined and useful concepts for the calculation of resolution limit for $\chi_{{\text{r}}}^{\left( i \right)} \left( {\varvec{x}} \right)$ and $\chi_{{\text{i}}}^{\left( i \right)} \left( {\varvec{x}} \right)$, respectively. Irrespective of the presence of the OTF, we can still use the 3-D aperture to evaluate and compare the resolution limits of all types of microscopy including the systems in the absence of LO in which OTFs cannot be defined.

**Figure 1:**
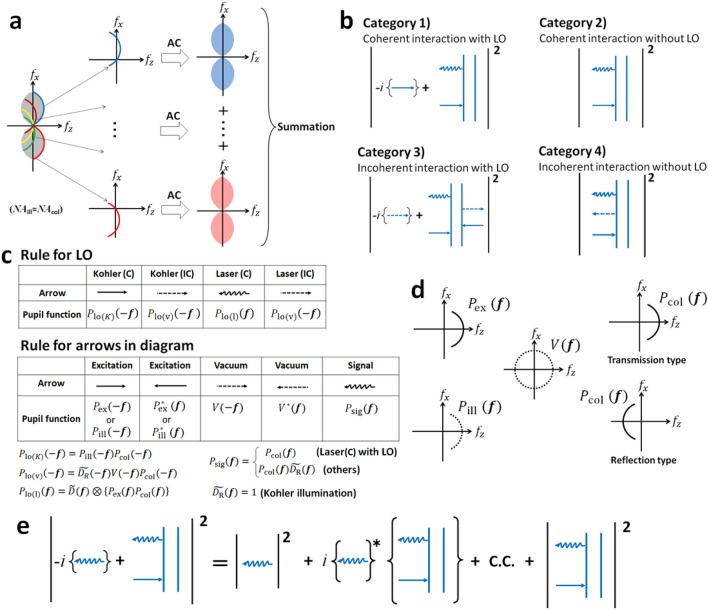
Diagram descriptions for the 3-D aperture. (**a**) The image formation properties of partially coherent bright-field microscopy in the frequency domain. A leftmost illustration describes the 3-D aperture. The autocorrelation (AC) of the 3-D aperture does not increase beyond the frequency cutoff of the 3-D aperture. Each arc of a different colour corresponds to the 3-D pupil function of the signal-collection system shifted depending on the position of the incoherent surface light source. Each arc does not interfere with each other. (**b**) Four types of Feynman diagrams for category (1)–(4). Each category shows an example: (1) linear absorption with the local oscillator (LO), (2) linear absorption in a partially coherent system without LO, (3) fluorescence, and (4) spontaneous parametric down-conversion (SPDC). (**c**) Correspondence rule between arrows and pupil functions for the LO and for the Feynman diagram. In Rule for LO, Kohler and laser denote Kohler illumination and laser microscopy, respectively, and C and IC represent coherent and incoherent interactions, respectively. (**d**) Diagrammatic illustration of pupil functions. The dotted curve represents the random phase. (**e**) Diagram expression of laser microscopy in Category (1), i.e., coherent light-matter interaction with the LO. This is an example of linear absorption with the LO. For convenience, the arrow of LO is changed to a wavy arrow.

Next, we consider the microscopy in the absence of the LO. The Fourier transform of Eq. (7) is given by

10 $$ \tilde{I}\left( {\varvec{f}} \right) = {\text{AC}}\left[ {\widetilde{{\chi^{\left( i \right)} }}\left( {\varvec{f}} \right)A\left( {\varvec{f}} \right)} \right], $$

where AC[$\cdots$] represents the autocorrelation. In the absence of the LO, the interference between the signal fields contributes to the image formation, which means that the OTF cannot be defined, resulting in the image that includes the undesired Fourier components that object does not have. However, even in this case, the 3-D aperture is the most appropriate criterion for obtaining the resolution limit, because the spatial frequency outside the 3-D aperture $A\left( {\varvec{f}} \right)$ can never be acquired. For example, we consider the 3-D aperture of bright-field microscopy. By performing a Fourier transform on Eq. (3), we obtain

11 $$ \widetilde{{I_{{\text{K}}} }}\left( {\varvec{f}} \right) = {\text{AC}}\left[ {\widetilde{{\chi^{\left( 1 \right)} }}\left( {\varvec{f}} \right)\left\{ {P_{{{\text{ill}}}} \left( { - {\varvec{f}}} \right) \otimes P_{{{\text{col}}}} \left( {\varvec{f}} \right)} \right\}} \right], $$

where the symbol $\otimes$ denotes the convolution, and $P_{{{\text{col}}}} \left( {\varvec{f}} \right)$ is a pupil function of the signal-collection system. Note that because $P_{{{\text{ill}}}} \left( {\varvec{f}} \right)$ possesses a random phase distribution, $\widetilde{{I_{K} }}\left( {\varvec{f}} \right)$ becomes

12 $$ \widetilde{{I_{{\text{K}}} }}\left( {\varvec{f}} \right) = \int {\text{AC}}\left[ {\widetilde{{\chi^{\left( 1 \right)} }}\left( {\varvec{f}} \right)P_{{{\text{col}}}} \left( {{\varvec{f}} - {\varvec{f}}_{{{\text{ill}}}} } \right)} \right]P_{{{\text{ill}}}} \left( {{\varvec{f}}_{{{\text{ill}}}} } \right)d^{3} {\varvec{f}}_{{{\text{ill}}}} , $$

by which one can identify the key terms of the partially coherent image formation^26^. Figure 1a shows the 3-D aperture of bright-field microscopy $A\left( {\varvec{f}} \right) = P_{{{\text{ill}}}} \left( { - {\varvec{f}}} \right) \otimes P_{{{\text{col}}}} \left( {\varvec{f}} \right)$. Each point of the incoherent surface light source, corresponding to a single point on $P_{{{\text{ill}}}} \left( {\varvec{f}} \right)$, produces an image, and the summation of all the images formed by each point of the light source provides the final image ${\text{I}}_{{\text{K}}} \left( { - \varvec{x}^{\prime}} \right)$. Due to the random phase distribution of $P_{{{\text{ill}}}} \left( {\varvec{f}} \right)$, the nonzero region of $\widetilde{{I_{{\text{K}}} }}\left( {\varvec{f}} \right)$ becomes equivalent to that of the 3-D aperture, which results in an unfilled region known as a missing cone^27^. In order to describe the resolution limits for all optical microscopy, we define the 3-D aperture as the most fundamental quantity. One can always use the 3-D aperture for comparing and evaluating the resolution limits, irrespective of the forms of microscopy and the presence or absence of the LO.

### Classification of optical microscopy

In order to find the formulas for the 3-D aperture calculation, we categorize optical microscopy into four types, i.e., 1) coherent systems with the LO, 2) coherent systems without the LO, 3) incoherent systems with the LO (e.g. fluorescence), and 4) incoherent systems without the LO (e.g. spontaneous parametric down-conversion (SPDC)^28^). In an incoherent system, the vacuum field is one of the excitation fields^29^, while in coherent system, all the excitation fields are real fields from the light sources. Figure 1b shows examples of these four types, where the LO is denoted by an arrow. In every case, the 3-D aperture is calculated by performing a Fourier transform of $h_{{\text{t}}} \left( {\varvec{x}} \right)$ or $h_{{\text{t}}} ^{\prime}\left( {\varvec{x}} \right)$. Through the Fourier transform of $h_{{\text{t}}} \left( {\varvec{x}} \right)$ or $h_{{\text{t}}} ^{\prime}\left( {\varvec{x}} \right)$, we can obtain a 3-D aperture calculation rule associated with the Feynman diagram that describes each light-matter interaction. We formulate this rule with respect to each type of microscope system after converting the q-numbers into c-numbers. As a result, we develop a general rule applicable to all microscope systems by associating the arrows in both the Feynman diagram and the LO with pupil functions as shown in Fig. 1c; note that some arrows also include the detector function. The descriptions of the pupil functions are given in Fig. 1d. Figure 1e shows the Feynman diagram representation of laser microscopy with coherent interaction, where for convenience, the arrow of the LO (excitation beam) is represented by the same wavy arrow as the signal field. When the LO is absent, the 3-D aperture can be calculated by connecting all the functions corresponding to each arrow in a diagram. When the LO is present, the 3-D aperture can be found by connecting all the functions with convolution, after first multiplying the complex conjugate of the LO function by the function of the identically-shaped arrow in the diagram.

We show examples of how to calculate the 3-D apertures for the four types of optical microscopy: category (1)–(4). The procedure to do this is detailed in the Supplementary Information. With respect to the detector size effect on the optical resolution, we address only laser microscopy that employs a single-pixel photodetector because we assume the pixel size of the 2-D detector in wide-field microscopy, such as the Kohler illumination system, to be sufficiently small. All types contain both Kohler illumination and laser microscopy. Hereafter, in all cases we assume that the detector is placed at the image conjugate plane.

### Potential capability of each light-matter interaction

**Figure 2:**
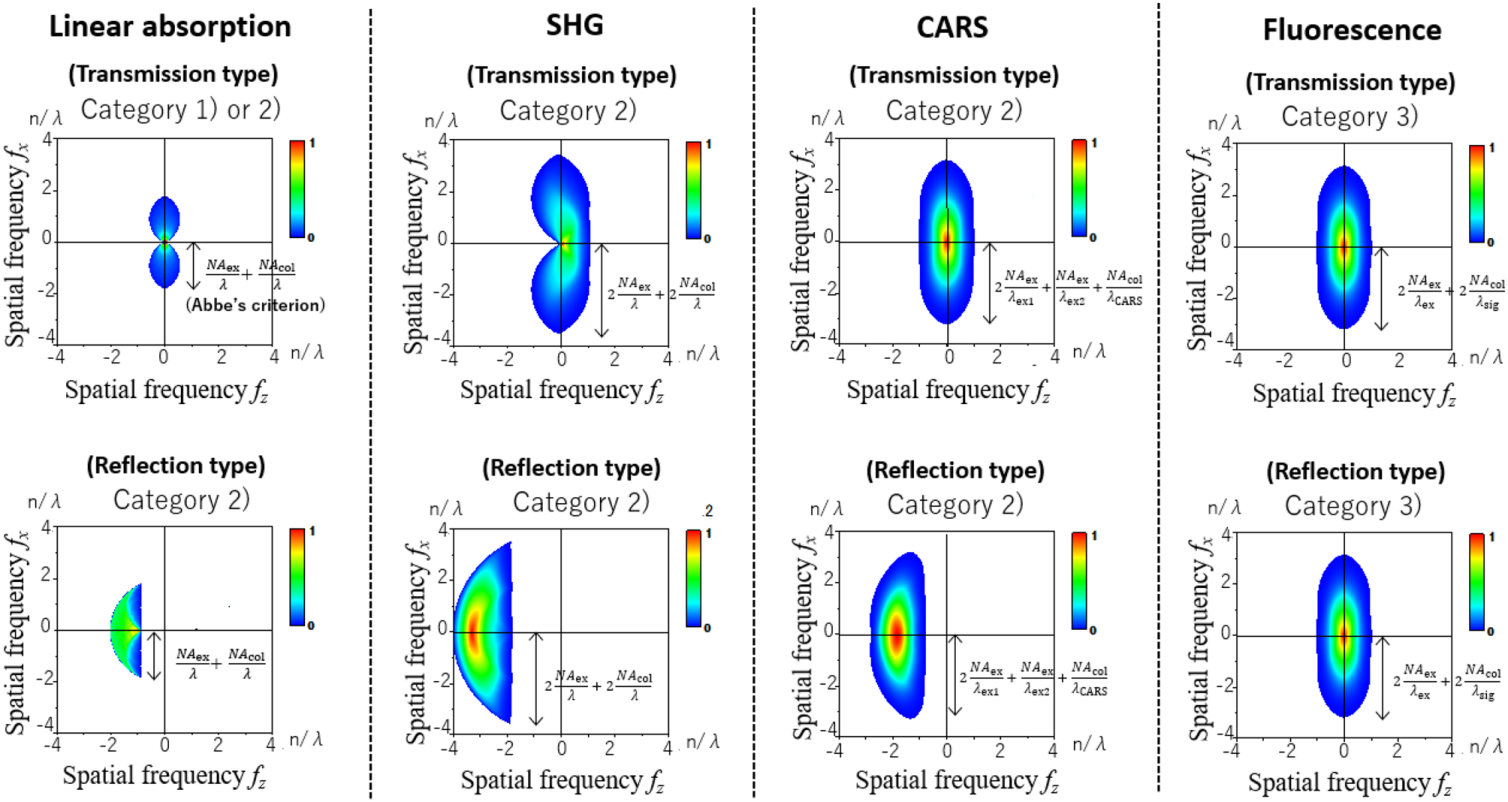
3-D apertures of confocal microscopy with typical light-matter interactions: linear absorption (LA), second harmonic generation (SHG), coherent anti-Stokes Raman scattering (CARS), and fluorescence. The upper and lower rows are for transmission and reflection types, respectively. Only the $\chi^{\left( 1 \right)}$-derived interaction including linear absorption obeys Abbe’s criterion 2*NA*/${\uplambda }$. Bright-field microscopy with intense non-diffracted light (LO), which belongs to category (1), shows the same 3-D aperture as the top-left one. All panels show the $f_{x}$-$f_{z}$ cross-sections, and they are rotationally symmetric around $f_{z}$ axis. The scale bars to the right of each panel indicate the frequency cutoff in $f_{x}$-$f_{y}$ directions as a guide. *n* is the average refractive index in the sample. In LA and SHG, ${\uplambda }$ is the wavelengths of the excitation beam. In CARS, $\lambda_{{{\text{ex}}1}}$ and $\lambda_{{{\text{ex}}2}}$ are the wavelengths of the first (pump) and second (Stokes) excitation beams, and $1/\lambda_{{{\text{CARS}}}} = 2/\lambda_{{{\text{ex}}1}} - 1/\lambda_{{{\text{ex}}2}}$. In fluorescence, $\lambda_{{{\text{ex}}}}$ and $\lambda_{{{\text{sig}}}}$ are the wavelengths of the excitation beam and signal. The numerical apertures of the excitation and signal-correction systems are $NA_{{{\text{ex}}}} = NA_{{{\text{col}}}} = 1.2$.

As an example of the system that draws out a maximum frequency cutoff, we take confocal microscopy with a confocal pinhole placed just before the detector, which corresponds to a sufficiently small detector: $D\left( {{\varvec{x}}_{{\text{d}}} } \right) = \delta \left( {{\varvec{x}}_{{\text{d}}} } \right)$. Confocal microscopy with any types of light-matter interactions categorized into 1) ~ 4) is one of the systems that exhibits the largest frequency cutoff of the 3-D aperture for a given interaction of interest. By substituting $D\left( {{\varvec{x}}_{{\text{d}}} } \right) = \left| {D_{{\text{R}}} \left( {{\varvec{x}}_{{\text{d}}} } \right)} \right|^{2} = \delta \left( {{\varvec{x}}_{{\text{d}}} } \right)$ into the detector function in Fig. 1c, we obtain Table 1, which shows the correspondence rule between the arrow and the pupil function for confocal microscopy. The 3-D aperture of confocal microscopy can be found by convolving all the pupil functions corresponding to the arrows in the relevant diagram. The 3-D apertures of confocal microscopy with typical light-matter interactions are shown in Fig. 2 (also see Supplementary Figure 1 and Supplementary Figure 2 for more detail). Note that in the reflection-type microscopy with coherent interaction, the LO usually does not exist, which means that the OTF cannot be defined. By contrast, in the reflection-type microscopy with incoherent interaction, the LO (which is the vacuum field in this case), inevitably exists, resulting in the presence of the OTF. This always becomes identical to that of the transmission type because the vacuum field modes in all directions exist around the sample.

We briefly refer to the difference between Kohler illumination and laser excitation microscopy (see also Supplementary information). In Kohler illumination systems with the LO, the image can be written as $\int K\left( {\varvec{x}} \right)h_{{{\text{col}}}} \left( { - {\varvec{x}}} \right)d^{3} {\varvec{x}}$ (see Eqs. (3) and (5)), resulting in $h_{{\text{t}}} \left( { - {\varvec{x}}} \right) = \left\{ {{\Gamma }_{12} \left( {\varvec{x}} \right) \otimes h_{{{\text{col}}}}^{*} \left( {\varvec{x}} \right)} \right\}h_{{{\text{col}}}} \left( { - {\varvec{x}}} \right)$. Accordingly, the Fourier transform of $h_{{\text{t}}} \left( {\varvec{x}} \right)$, i.e., the 3-D aperture becomes $\left[ {\left| {P_{{{\text{ill}}}} \left( { - {\varvec{f}}} \right)} \right|^{2} \left\{ {P_{{{\text{col}}}} \left( { - {\varvec{f}}} \right)} \right\}^{*} } \right] \otimes P_{{{\text{col}}}} \left( {\varvec{f}} \right)$, whose shape is equal to that of the transmission-type confocal microscopy with χ^(1)^-derived interactions under the same NA condition, shown at the top-left section in Fig. 2. In Kohler illumination systems without the LO, the 3-D aperture becomes $P_{{{\text{ill}}}} \left( { - {\varvec{f}}} \right) \otimes P_{{{\text{col}}}} \left( {\varvec{f}} \right)$ as shown at the leftmost section in Fig. 1a. Irrespective of the presence or absence of the LO, the frequency cutoffs of 3-D apertures are identical, which means that the highest frequencies of a grating structure in the sample that can be resolved are equal. Also, for a single point object, the images in both cases become the same. By contrast, in laser excitation microscopy, despite the same highest resolvable frequencies regardless of the LO, the images of a single point are different depending on the presence or absence of the LO. Although the image of a single point in laser microscopy without the LO appears to be smaller than that of the system with the LO as expressed by Eqs. (8) and (10), a single point cannot become a criterion for resolution power. Since only the frequencies inside the 3-D aperture can contribute to the image formation, the 3-D apertures determine the resolution limits for all forms of microscopy.

The 3-D apertures in Fig. 2 (also Supplementary Figures 1 and 2) indicate the theoretical limits of optical resolutions. However, in practical systems, signal-to-noise ratio (SNR) also influences the image quality. In most experiments, the signal frequencies in the blue regions of 3-D apertures are more likely to be buried in noise, which causes an effective frequency cutoff to be smaller than the theoretical limit. In the high-speed imaging systems that does not obtain an enough brightness in image, the effective frequency cutoff might approach a border between the blue and green regions in 3-D apertures. An exception is the optical microscopy using χ^(1)^-derived interactions, which has a sufficiently intense signal. Note that finite pinhole size also has an effect of decrease in the frequency cutoff particularly in incoherent interactions, but in this case the theoretical limit lies in the vicinity of the border mentioned above and the effective frequency cutoff with a finite SNR is also nearly equal to the border. Even if an imaging system is perfectly set up and devised to obtain a high SNR, the system cannot observe the Fourier components outside the frequency cutoff determined by the light-matter interaction used. Thus, the theoretical limits are still useful for the microscopy development. Table 2 illustrates a list comparing the theoretical frequency cutoffs of typical microscopy. Each arrow in a Feynman diagram corresponds to ${\text{NA}}/{\uplambda }$, and the frequency cutoff in x–y direction is found by summing them, where the wavelength and NA for each arrow must be considered.

**Table 2:**
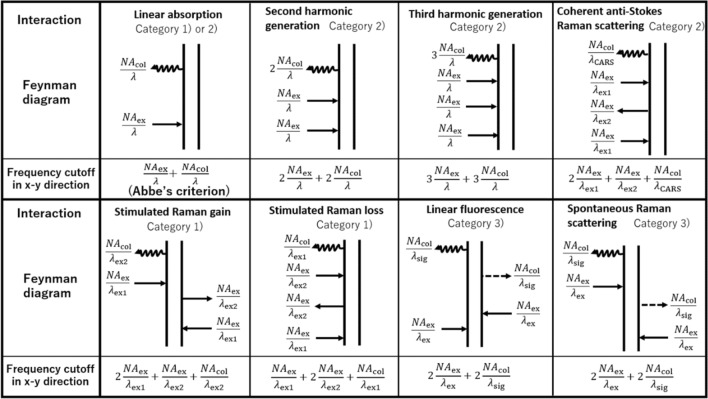
A comparison table of the maximum frequency cutoff in x–y direction that each light-matter interaction intrinsically possesses.

Some typical interactions are shown with Feynman diagrams. For $\lambda_{{{\text{ex}}}}$ and $\lambda_{{{\text{sig}}}}$, etc., see the legend of Fig. 2.

## Discussion

In this work, we have extended the concept of the diffraction limit to address all types of microscopy with the full range of light-matter interactions, including linear, nonlinear, coherent, and incoherent phenomena, and irrespective of the presence of the LO. We have integrated both Kohler illumination and laser excitation microscopy into a single theory in order to determine the optical resolution of all microscopy techniques in the same framework. Interestingly, the dotted arrows in Table 1 imply that the vacuum field improves the resolution limit in incoherent interactions. Our framework is formulated by combining techniques from classical, nonlinear, and quantum optics in order to generalize the calculation rules of the resolution limit, and to unify the image-formation formulas. Moreover, our diagrammatic methodology provides a bird’s-eye view of microscopy classification that intuitively reveals the relationships between the light-matter interactions and the resolution limits. This insight into the origin of the resolution limit can connect imaging systems from a variety of disciplines.

**Figure 3:**
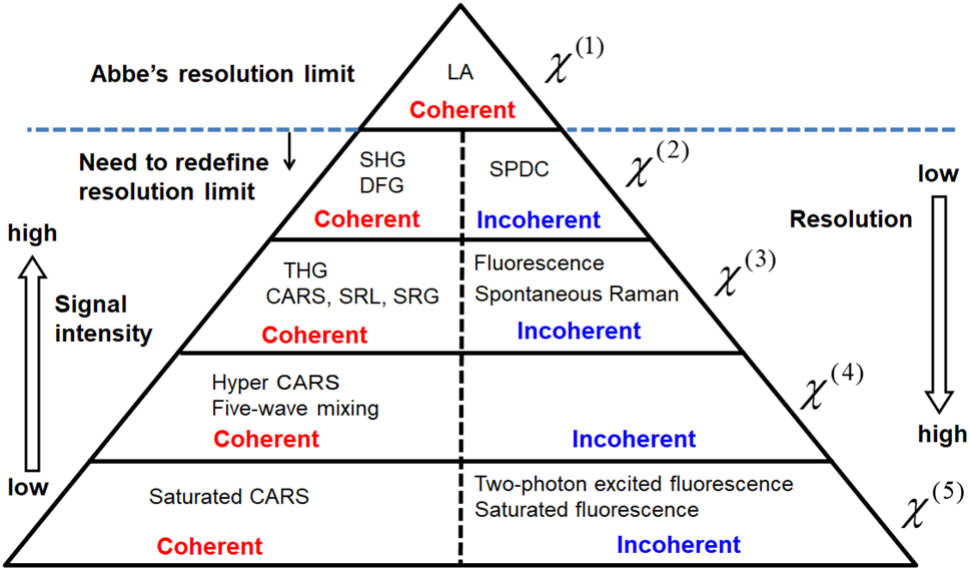
Classification of light-matter interactions. Only the $\chi^{\left( 1 \right)}$-derived interaction obeys Abbe’s resolution limit 2*NA*/${\uplambda }$. Interactions of a higher-order than $\chi^{\left( 1 \right)}$ have both coherent and incoherent light-matter interactions. Not all interactions are shown here. LA: linear absorption, SHG: second harmonic generation, DFG: difference frequency generation, THG: third harmonic generation, CARS: coherent anti-Stokes Raman scattering, SRG: stimulated Raman gain, SRL: stimulated Raman loss, SPDC: spontaneous parametric down-conversion.

The double-sided Feynman diagram was originally invented to categorize all light-matter interactions and calculate the amplitude and phase of ${\upchi }^{\left( i \right)}$. In this study, we have added one more capability to the Feynman diagram: the calculation of the resolution limit of optical microscopy. We have also defined the 3-D aperture, which can be found by the Feynman diagram, so as to evaluate and compare the resolution limits of all types of optical microscopy. The Fourier components outside of the 3-D aperture do not contribute to the image formation, and the OTF can be defined only if the LO exists, while the 3-D aperture can always be defined. Therefore, the 3-D aperture is inevitably the best indicator of the optical resolution, especially for cases when the OTF is not defined. Figure 3 shows a pyramid for the classification of interactions. All interactions contain both coherent and incoherent light-matter interactions, except for $\chi^{\left( 1 \right)}$-derived interaction, which has only a coherent interaction. For $\chi^{\left( 1 \right)}$-derived interaction, the resolution limit obeys Abbe’s law. By contrast, for all other $\chi^{\left( i \right)}$-derived interactions (*i*≧2), including fluorescence, the resolution limit is higher than Abbe’s law and follows the hierarchy outlined in the pyramid.

In our framework, cutting-edge microscopy such as structured illumination microscopy (SIM)^30^, image scanning microscopy (ISM)^31^, and stimulated emission depletion microscopy (STED)^32^, can also be addressed by considering the pupil apodization, plural-order interactions occurring simultaneously, and so on. While SIM and ISM can be treated by applying the apodization of the illumination system and the detector-function characteristic, respectively, the resolution limits of both techniques cannot surpass the theoretical limitation defined by fluorescence. In STED, in addition to the apodization of excitation beam, plural-order diagrams ($\chi^{\left( 3 \right)}$-fluorescence, $\chi^{\left( 5 \right)}$-saturated fluorescence, $\chi^{\left( 7 \right)}$-higher-order saturated fluorescence,…) contribute to the image formation, but the highest-order diagram that is not buried in noise determines the effective frequency cutoff. Our resolution limit rule does not include near-field microscopy, but its image-forming properties can be treated by considering the signal field $\hat{a}_{{{\text{sig}}}}^{ + } \left( {\varvec{x}} \right)$ that is created at a single point ${\varvec{x}}$ and propagates to a detecting point ${\varvec{x}}_{{\text{d}}}$, where it is annihilated by $\hat{a}_{{\det}} \left( {{\varvec{x}}_{{\text{d}}} } \right)$ instead of by $\hat{a}_{{{\text{col}}}} \left( {{\varvec{x}}_{{\text{d}}} } \right)$. The typical exception to our theory concerning far-field microscopy involves localization microscopy such as PALM^33^ and STORM^34^, which assume a priori information about the object, i.e. that the sample is composed of sparsely distributed isolated points. Although OCT^9^ basically follows Abbe’s law, we need to expand 3-D aperture into four-dimensional aperture to precisely discuss it, which will be a next step.

## Methods

### Double-sided Feynman diagram

Double-sided Feynman diagrams have long been used in nonlinear spectroscopies, focusing on time/frequency dual variables^19^. In previous theoretical work^35^, X-ray 3-D diffraction pattern is studied using Feynman diagrams, where the results are illustrated with time- and wavevector-resolved signals. In this work, we also use double-sided Feynman diagrams to describe light-matter interactions. To address microscopy with high NA objective lens, we connect Feynman diagram with the 3-D aperture defined by the pupil functions that limit the wavevectors.

We briefly review Feynman diagram. In the diagram, a density matrix of a molecular ensemble evolves together with the excitation and signal electric fields that move from the bottom towards the top of the diagram over time, as shown in Supplementary Figure 4a. In general, the interaction of interest is expressed by multiple simultaneous diagrams including the permutation of the excitation fields, but they all represent the same optical resolution. Therefore, we represent an interaction using only one diagram. In the diagram, the lateral arrows indicate electric fields, and the left and right vertical lines represent the ket and bra sides of the density matrix, respectively. As a simpler way to understand the physical picture of the diagram, we can focus on a single molecule in the ensemble, where the wave function changes during each interaction with the excitation and signal electric fields. At any given moment, the wave function is a superposition between the states represented by the ket and bra sides.

### Light-matter interactions in free space

We analyse the fundamental mathematics underlying the physical phenomena in a united fashion by using quantum-optical notation^36^ to address all light-matter interactions, including incoherent interactions for which a vacuum field is responsible. In quantum optics, electric fields are replaced by annihilation/creation operators into which the vacuum field is incorporated. Before dealing with microscopy, we first analyse the simple case where the creation and annihilation of excitation photons occurs in free space along with the transitions of molecules (including virtual transitions). This results in the creation of a signal photon from the molecule that is annihilated at the detection position ***x***_d_ = (*x*_d_, *y*_d_, *z*_d_), as shown in Supplementary Figure 4b. To be more exact, we consider the molecular ensemble with nonlinear susceptibility χ^(*i*)^ instead of a single molecule. In a Feynman diagram, the left- and right-pointing arrows correspond to creation operators *â*^+^(***x***) and annihilation operators *â*(***x***), respectively. Because solid arrows are generally used to represent the excitation field (real photons) in a diagram, we use a dotted arrow to represent the vacuum field. We draw wavy arrows for the signal field, in which the arrow for the signal invariably emerges from the ket side to avoid redundant elements addition (Supplementary Figure 4c). A wavy arrow corresponds to the signal creation operator $\hat{a}_{{{\text{sig}}}}^{ + } \left( {\varvec{x}} \right)$. We define the interaction operator Ê_int_(***x***) as the product of all the operators in the diagram of interest with the excitation operator $\hat{E}_{{{\text{ex}}}} \left( {\varvec{x}} \right)$, i.e., $\hat{E}_{{{\text{int}}}} \left( {\varvec{x}} \right) = \hat{E}_{{{\text{ex}}}} \left( {\varvec{x}} \right)\hat{a}_{{{\text{sig}}}}^{ + } \left( {\varvec{x}} \right)$. The excitation operator for the *i*-th order light-matter interaction is composed of *i* creation/annihilation operators. Some diagrams have an inner dotted line indicating the longitudinal relaxation, but it does not influence the optical resolution and does not relate to the interaction operator. Only electric fields corresponding to arrows form the ASFs that determine the optical resolution.

We assume that the laser beam incident on the molecular ensemble is in a coherent state $\left| \alpha \right\rangle$. In this formulation, we also incorporate the vacuum state around the sample $\left| 0 \right\rangle$ (see Supplementary Equation (3)). Under the excitation condition $\left| \alpha \right\rangle \left| 0 \right\rangle$, the expected amplitude of the signal that was created at ***x*** and detected at ***x***_d_ can be expressed as $\left\langle 0 \right|\left\langle \alpha \right|\chi^{\left( i \right)} {\hat{\text{E}}}_{{{\text{int}}}} \left( {\varvec{x}} \right){ }\hat{a}_{{\det}} \left( {{\varvec{x}}_{{\text{d}}} } \right)\left| \alpha \right\rangle \left| 0 \right\rangle$, where we define an annihilation operator $\hat{a}_{{\det}} \left( {{\varvec{x}}_{{\text{d}}} } \right)$ that acts in free space without lenses (see Supplementary Equation (13)). For example, in linear absorption, by using the equation Ê_int_(***x***) = $\hat{a}\left( {\varvec{x}} \right)\hat{a}_{{{\text{sig}}}}^{ + } \left( {\varvec{x}} \right)$ with $\hat{a}\left( {\varvec{x}} \right)$ acting on $\left| \alpha \right\rangle$ and $\hat{a}_{{{\text{sig}}}}^{ + } \left( {\varvec{x}} \right)$ acting on $\left| 0 \right\rangle$, and the normalizations $\left\langle {\alpha {|}\alpha } \right\rangle = 1$ and $\left\langle {0{|}0} \right\rangle = 1$, the expected amplitude becomes

13 $$ \begin{aligned} & \left\langle 0 \right|\left\langle \alpha \right| \vdots \chi^{\left( 1 \right)} \hat{a}\left( {\varvec{x}} \right)\hat{a}_{{\det}} \left( {{\varvec{x}}_{{\text{d}}} } \right)\hat{a}_{{{\text{sig}}}}^{ + } \left( {\varvec{x}} \right) \vdots \left| \alpha \right\rangle \left| 0 \right\rangle \\ & \quad = \chi^{\left( 1 \right)} \left\langle \alpha \right|\hat{a}\left( {\varvec{x}} \right)\left| \alpha \right\rangle \left\langle 0 \right|\hat{a}_{{\det}} \left( {{\varvec{x}}_{{\text{d}}} } \right)\hat{a}_{{{\text{sig}}}}^{ + } \left( {\varvec{x}} \right)\left| 0 \right\rangle \\ & \quad = \chi^{\left( 1 \right)} \alpha \left( {\varvec{x}} \right)G\left( {{\varvec{x}}_{{\text{d}}} - {\varvec{x}}} \right) \\ \end{aligned} $$

where a symbol $\vdots { }\, \vdots$ represents the ordering rule of operators defined in the next Section, $G\left( {{\varvec{x}}_{{\text{d}}} - {\varvec{x}}} \right)$ denotes the Green’s function for the signal photon propagating from ***x*** to ***x***_d_, and ${\upalpha }\left( {\varvec{x}} \right)$ is the complex function obtained from the equation $\hat{a}\left( {\varvec{x}} \right)\left| \alpha \right\rangle = \alpha \left( {\varvec{x}} \right)\left| \alpha \right\rangle$. The intensity is found by taking the square of the modulus of Eq. (13).

### Ordering rule of operators

We now establish the operator ordering so that we can then ignore the vacuum fluctuation included in the light source, and can extract the vacuum effect only when the vacuum field directly contributes to the Green’s function such as $\left\langle 0 \right|\hat{a}_{{\det}} \left( {{\varvec{x}}_{{\text{d}}} } \right)a_{{{\text{sig}}}}^{ + } \left( {\varvec{x}} \right)\left| 0 \right\rangle$. This operator ordering allows for the discussion of the theoretical resolution limit. The operators *â*_ex_(***x***) and *â*_lo(l)_(***x***_d_) act on $\left | \alpha \right \rangle_{{{\text{ex}}}}$, and the operators *â*_vac_(***x***), *â*_lo(v)_(***x***_d_), *â*_sig_(***x***), and *â*_col_(***x***_d_) act on $\left| 0 \right\rangle$ (See Supplementary Note 8). For the operator ordering, we introduce the symbol $\vdots { }\, \vdots$. In the area between the symbols $\vdots { }\, \vdots$, the operator order is rearranged as follows:

- Rule for the operators acting on $\left | \alpha \right \rangle_{{{\text{ex}}}}$
  Normal ordered product: creation operators are placed to the left of the annihilation operators in the product.
- Rule for the operators acting on $\left | 0 \right \rangle$
  Anti-normal ordered product: annihilation operators are placed to the left of the creation operators in the product.

The specially-ordered product defined above means that the vacuum field cannot be observed, except when considering the propagator represented as the vacuum expectation value, such as $\left\langle 0 \right|\hat{a}_{{{\text{col}}}} \left( {{\varvec{x}}_{{\text{d}}} } \right)\hat{a}_{{{\text{sig}}}}^{ + } \left( {\varvec{x}} \right)\left| 0 \right\rangle$. Note that we ignore the vacuum expectation value $\left\langle 0 \right|\hat{a}_{{{\text{lo}}\left( {\text{v}} \right)}} \left( {{\varvec{x}}_{{\text{d}}} } \right)\hat{a}_{{{\text{lo}}\left( {\text{v}} \right)}}^{ + } \left( {{\varvec{x}}_{{\text{d}}} } \right)\left| 0 \right\rangle$, which cannot be observed in practical experiments.

### Quantum image formation theory of laser microscopy

In the more specific case of linear/nonlinear laser excitation microscopy, one or two excitation beams are usually employed to generate the signal. We define the excitation condition $\left | \alpha \right \rangle_{{{\text{ex}}}}$ as the state representing all laser beams focused onto the sample (see Supplementary Note 6). We also define the annihilation operator $\hat{a}_{{{\text{col}}}} \left( {{\varvec{x}}_{{\text{d}}} } \right)$ at the microscopy detecting point ${\varvec{x}}_{{\text{d}}}$. The creation *â*^+^(***x***) and annihilation *â*(***x***) operators for the excitation and signal fields in real space, respectively, are found from the inverse Fourier transform of the product of the 3-D pupil function, *P*(***f***), and the operators in wavenumber space, *â*^+^(***f)*** or *â*(***f)*** (see Supplementary Equations (6)–(12)). To generalize these image formation theories, we replace $\chi^{\left( 1 \right)} \left( {\varvec{x}} \right)$ in Eq. (2) with $\chi^{\left( i \right)} \left( {\varvec{x}} \right)$ and the electric fields with the annihilation operators. The image intensity ${\text{I}}\left( {\varvec{x}^{\prime}} \right)$ at the sample-stage displacement ${\varvec{x}}^{\prime } = \left( {x^{\prime } ,y^{\prime } ,z^{\prime } } \right)$ is given by^18^

14 $$ \left\langle 0 \right|_{{{\text{ex}}}} \left\langle \alpha \right| \vdots \int \left| { - i\hat{a}_{{{\text{lo}}}} \left( {{\varvec{x}}_{{\text{d}}} } \right) + \int \chi^{\left( i \right)} \left( {{\varvec{x}} - \varvec{x}^{\prime}} \right)\hat{E}_{{{\text{int}}}} \left( {\varvec{x}} \right)\hat{a}_{{{\text{col}}}} \left( {{\varvec{x}}_{{\text{d}}} } \right)d^{3} {\varvec{x}} } \right|^{2} D\left( {{\varvec{x}}_{{\text{d}}} } \right)d^{3} {\varvec{x}}_{{\text{d}}} \vdots \left| \alpha \right\rangle_{{{\text{ex}}}} \left| 0 \right\rangle , $$

where $\left| \alpha \right\rangle_{{{\text{ex}}}} \left| 0 \right\rangle$ corresponds to the excitation condition $\left| {{\text{ex}}} \right\rangle$, and $\hat{a}_{{{\text{lo}}}} \left( {{\varvec{x}}_{{\text{d}}} } \right)$ is the operator for the LO.

In some types of microscopy, the excitation light from the excitation system or the vacuum field around the sample acts as the LO, and it interferes with the signal field at the detecting point. The vacuum field causes incoherent interactions and it becomes the LO in some interactions, including fluorescence and spontaneous Raman scattering. Note that the vacuum field itself cannot be observed^36^. If the LO is not present, the three terms that include $\hat{a}_{{{\text{lo}}}} \left( {{\varvec{x}}_{{\text{d}}} } \right)$ vanish after the square of the modulus, $\left\langle 0 \right|$, and $\left| 0 \right\rangle$ in Eq. (14) are expanded. Only the fourth term remains, and this term corresponds to the square of the modulus of the signal field and does not include $\hat{a}_{{{\text{lo}}}} \left( {{\varvec{x}}_{{\text{d}}} } \right)$. In this case, the fourth term is responsible for the resolution limit.

If the LO is present, the fourth term is negligible and the cross terms (the second and third terms) determine the resolution limit. Because the $\hat{E}_{{{\text{ex}}}} \left( {\varvec{x}} \right)$ operator is composed of the product of all the operators in the diagram of interest except for $\hat{a}_{{{\text{sig}}}}^{ + } \left( {\varvec{x}} \right)$, as mentioned above, in microscopy with the LO, the image intensity becomes

15 $$ \begin{aligned} I\left( {{\varvec{x}}^{\prime}} \right) & = i\int \int \chi^{\left( i \right)} \left( {{\varvec{x}} - \varvec{x}^{\prime}} \right)\left\langle 0 \right|_{{{\text{ex}}}} \left\langle \alpha \right| \vdots \hat{E}_{{{\text{ex}}}} \left( {\varvec{x}} \right)\hat{a}_{{{\text{sig}}}}^{ + } \left( {\varvec{x}} \right)\hat{a}_{{{\text{col}}}} \left( {{\varvec{x}}_{{\text{d}}} } \right)\hat{a}_{{{\text{lo}}}}^{ + } \left( {{\varvec{x}}_{{\text{d}}} } \right) \vdots \left| \alpha \right\rangle_{{{\text{ex}}}} \left| 0 \right\rangle d^{3} \varvec{x}D\left( {{\varvec{x}}_{{\text{d}}} } \right)d^{3} {\varvec{x}}_{{\text{d}}} \\ & \quad + c.c.{ }, \\ \end{aligned} $$

where c.c. denotes complex conjugate, the fourth term is ignored, and the first term is omitted that is not responsible for image shape. Note that the signal field is created from the vacuum field by $\hat{a}_{{{\text{sig}}}}^{ + } \left( {\varvec{x}} \right)$, and $\hat{a}_{{{\text{col}}}} \left( {{\varvec{x}}_{{\text{d}}} } \right)$ also acts on the vacuum field. The vacuum field operator acts on $\left| 0 \right\rangle$ regardless of whether the operator corresponds to the LO field or one of the excitation fields. Likewise, the laser field operator acts on $\left| \alpha \right\rangle_{{{\text{ex}}}}$ irrespective of whether the operator corresponds to the LO or the excitation field.

In microscopy without the LO, the expected value of the image intensity is

16 $$ \begin{aligned}   I\left( {\varvec{x}^{\prime } } \right) &  = \int \left| {\int \chi ^{{\left( i \right)}} \left( {\varvec{x} - \varvec{x}^{\prime } } \right)\left| 0 \right\rangle _{{{\text{ex}}}} \,\left\langle \alpha  \right| \vdots \hat{E}_{{{\text{ex}}}} \left( \varvec{x} \right)~~\hat{a}_{{{\text{col}}}} \left( {\varvec{x}_{{\text{d}}} } \right)\hat{a}_{{{\text{sig}}}}^{ + } \left( \varvec{x} \right) \vdots \left| \alpha  \right\rangle _{{{\text{ex}}}} \,\left| 0 \right\rangle d^{3} \varvec{x}} \right|^{2} D\left( {\varvec{x}_{{\text{d}}} } \right)d^{3} \varvec{x}_{{\text{d}}}  \\     & \quad  = \int \int\int \chi ^{{\left( i \right)}} \left( {\varvec{x}_{1}  - \varvec{x}^{\prime } } \right)\left\{ {\chi ^{{\left( i \right)}} \left( {\varvec{x}_{2}  - \varvec{x}^{\prime } } \right)} \right\}^{*} \left\langle 0 \right|\,{_{{\text{ex}}}}\left\langle \alpha  \right| \\     & \quad  \times ~ \vdots \hat{E}_{{{\text{ex}}}} \left( {\varvec{x}_{1} } \right)\hat{a}_{{{\text{sig}}}}^{ + } \left( {\varvec{x}_{1} } \right)\hat{a}_{{{\text{col}}}} \left( {\varvec{x}_{{\text{d}}} } \right)\hat{E}_{{{\text{ex}}}}^{ + } \left( {\varvec{x}_{2} } \right)\hat{a}_{{{\text{sig}}}} \left( {\varvec{x}_{2} } \right)\hat{a}_{{{\text{col}}}}^{ + } \left( {\varvec{x}_{{\text{d}}} } \right) \vdots  \\     & \quad  \times \left| \alpha  \right\rangle _{{{\text{ex}}}} \,\left| 0 \right\rangle d^{3} \varvec{x}_{1} d^{3} \varvec{x}_{2} D\left( {\varvec{x}_{{\text{d}}} } \right)d^{3} \varvec{x}_{{\text{d}}} . \\  \end{aligned}   $$

Here, we employ the formula for the vacuum expectation value in frequency domain (vacuum formula), e.g., $\left\langle 0 \right|\hat{a}_{1} \left( {{\varvec{f}}_{1} } \right)\hat{a}_{2} \left( {{\varvec{f}}_{2} } \right)\hat{a}_{3}^{ + } \left( {{\varvec{f}}_{3} } \right)\hat{a}_{4}^{ + } \left( {{\varvec{f}}_{4} } \right)\left| 0 \right\rangle = \delta \left( {{\varvec{f}}_{1} - {\varvec{f}}_{3} } \right)\delta \left( {{\varvec{f}}_{2} - {\varvec{f}}_{4} } \right) + \delta \left( {{\varvec{f}}_{1} - {\varvec{f}}_{4} } \right)\delta \left( {{\varvec{f}}_{2} - {\varvec{f}}_{3} } \right)$, where $\delta \left( {\varvec{f}} \right)$ denotes Dirac’s delta function and takes only the one term that corresponds to an observable physical phenomenon. Note that the terms exist based on the number of combinations between the creation and annihilation operators. Only when the numbers of the creation and annihilation operators are equal, the vacuum expectation value remains.

### Conversion from q-number to c-number

Next, we convert the field operator (q-number) into a classical function (c-number). See also Supplementary Note 9. We start from the operator acting on the vacuum state $\left| 0 \right\rangle$. The signal emitted from a single point ***x*** in the sample forms the electric field distribution, i.e., the ASF, at the detection position ***x***_d_:

17 $$ h_{{{\text{col}}}} \left( {{\varvec{x}}_{{\text{d}}} - {\varvec{x}}} \right) = { }\left\langle 0 \right|\hat{a}_{{{\text{col}}}} \left( {{\varvec{x}}_{{\text{d}}} } \right)\hat{a}_{{{\text{sig}}}}^{ + } \left( {\varvec{x}} \right)\left| 0 \right\rangle , $$

which appears across all types of optical microscopy. Moreover, in an incoherent interaction, where the vacuum field acts as one of the excitation fields, the operator of the vacuum $\hat{a}_{{{\text{vac}}}} \left( {\varvec{x}} \right)$ in $\hat{E}_{{{\text{ex}}}} \left( {\varvec{x}} \right)$ acts on $\left| 0 \right\rangle$. Particularly when the LO is also the vacuum field, such as in fluorescence or spontaneous Raman scattering, a well-known expression emerges:

18 $$ \left\langle 0 \right|\hat{a}_{{{\text{vac}}}} \left( {\varvec{x}} \right)\hat{a}_{{{\text{col}}}} \left( {{\varvec{x}}_{{\text{d}}} } \right)\hat{a}_{{{\text{lo}}\left( {\text{v}} \right)}}^{ + } \left( {{\varvec{x}}_{{\text{d}}} } \right)\hat{a}_{{{\text{sig}}}}^{ + } \left( {\varvec{x}} \right)\left| 0 \right\rangle = \left\{ {h_{{{\text{col}}}} \left( {{\varvec{x}}_{{\text{d}}} - {\varvec{x}}} \right)} \right\}^{*} h_{{{\text{col}}}} \left( {{\varvec{x}}_{{\text{d}}} - {\varvec{x}}} \right). $$

The right-hand side of Eq. (18) corresponds to the point spread function (PSF) formed by the signal-collection system in fluorescence microscopy. In Eq. (18), we substitute $\hat{a}_{{{\text{lo}}}}^{ + }$ with $\hat{a}_{{{\text{lo}}\left( {\text{v}} \right)}}^{ + }$ in order to clarify which state the operator acts on. We also use the relation $\left\langle 0 \right|\hat{a}_{{{\text{vac}}}} \left( {\varvec{x}} \right)\hat{a}_{{{\text{lo}}\left( {\text{v}} \right)}}^{ + } \left( {{\varvec{x}}_{{\text{d}}} } \right)\left| 0 \right\rangle = \left\{ {\left\langle 0 \right|\hat{a}_{{{\text{lo}}\left( {\text{v}} \right)}} \left( {\varvec{x}} \right)\hat{a}_{{{\text{vac}}}}^{ + } \left( {{\varvec{x}}_{{\text{d}}} } \right)\left| 0 \right\rangle } \right\}^{*}$ after applying the same vacuum formula as used in Eq. (16).

We now also consider the operator acting on the coherent state for the excitation laser beam $\left | \alpha \right \rangle_{{{\text{ex}}}}$. For example, by using Eq. (15) and (18), we obtain the image intensity of fluorescence laser microscopy by observing the susceptibility distribution $\chi_{{{\text{flu}}}}^{\left( 3 \right)} \left( {\varvec{x}} \right)$:

19 $$ \begin{aligned} I\left( {{\varvec{x}}^{\prime}} \right) & = i\int \int \chi_{{{\text{flu}}}}^{\left( 3 \right)} \left( {{\varvec{x}} - \varvec{x}^{\prime}} \right) \,_{{{\text{ex}}}} \left\langle \alpha \right|\hat{a}_{{{\text{ex}}}}^{ + } \left( {\varvec{x}} \right)\hat{a}_{{{\text{ex}}}} \left( {\varvec{x}} \right)\left| \alpha \right\rangle_{{{\text{ex}}}} \left| {h_{{{\text{col}}}} \left( {{\varvec{x}}_{{\text{d}}} - {\varvec{x}}} \right)} \right|^{2} d^{3} \varvec{x}D\left( {{\varvec{x}}_{{\text{d}}} } \right)d^{3} {\varvec{x}}_{{\text{d}}} + c.c. \\ & = - 2\int \int {\text{Im}}\left[ {\chi_{{{\text{flu}}}}^{\left( 3 \right)} \left( {{\varvec{x}} - \varvec{x}^{\prime}} \right)} \right] \left| {\alpha_{{{\text{ex}}}} \left( {\varvec{x}} \right)} \right|^{2} \left| {h_{{{\text{col}}}} \left( {{\varvec{x}}_{{\text{d}}} - {\varvec{x}}} \right)} \right|^{2} d^{3} \varvec{x}D\left( {{\varvec{x}}_{{\text{d}}} } \right)d^{3} {\varvec{x}}_{{\text{d}}} , \\ \end{aligned} $$

where $\hat{a}_{{{\text{ex}}}} \left( {\varvec{x}} \right)$ is an annihilation operator representing the excitation laser beam focused on the sample, and we used $_{{{\text{ex}}}} \left\langle \alpha \right|\hat{a}_{{{\text{ex}}}}^{ + } \left( {\varvec{x}} \right)\hat{a}_{{{\text{ex}}}} \left( {\varvec{x}} \right)\left| \alpha \right\rangle_{{{\text{ex}}}} = \left| {\alpha_{{{\text{ex}}}} \left( {\varvec{x}} \right)} \right|^{2}$, which corresponds to a PSF formed by the excitation system. In fluorescence, the vacuum field acts as an LO, but the term corresponding to the square of the modulus of the vacuum field is omitted because it cannot be observed. Equation (19) matches a well-known phenomenologically derived image-forming formula for fluorescence laser microscopy^10^, if $- 2{\text{Im}}\left[ {\chi_{{{\text{flu}}}}^{\left( 3 \right)} \left( {\varvec{x}} \right)} \right]$, which is a positive function, is assumed to be the object.

## Supplementary information

Supplementary file1
