# Supplementary material for: A general theory of far-field optical microscopy image formation and resolution limit using double-sided Feynman diagrams
Source: Sci Rep. 2020 Oct 19;10:17644. doi: 10.1038/s41598-020-73584-1 (PMC7573692; doi:10.1038/s41598-020-73584-1)
Supplement: Supplementary file 1 — Supplementary file1 [file 41598_2020_73584_MOESM1_ESM.pdf]

Supplementary information

A general theory of far-field optical microscopy image formation and resolution limit using double-sided Feynman diagrams

NAOKI FUKUTAKE[1]

[1]*Nikon Corporation, 471 Nagaodai-cho, Sakae-ku, Yokohama-city, Kanagawa, 244-8533 Japan*

E-mail: Naoki.Fukutake@nikon.com

Supplementary Note 1: Category 1)

In category 1), a coherent system with the LO, we first consider laser microscopy with a finite-sized single-pixel photodetector as a typical example. For simplicity, we consider the easiest light-matter interaction: an $\chi^{(1)}$-derived interaction such as linear absorption. The diagram (including the LO) is shown in Fig. 1 (e) of the main manuscript. After multiplying $P_{\rm col}(\boldsymbol{f})$ of the signal by the complex conjugate of $\widetilde{D}(\boldsymbol{f}) \otimes \{P_{\rm ex}(\boldsymbol{f})P_{\rm col}(\boldsymbol{f})\}$ of the LO and convolving it with $P_{\rm ex}(-\boldsymbol{f})$ of the excitation field, the 3-D aperture is found to be $\left[\left\{\widetilde{D}(\boldsymbol{f}) \otimes (P_{\rm ex}(\boldsymbol{f})P_{\rm col}(\boldsymbol{f}))\right\}^* P_{\rm col}(\boldsymbol{f})\right] \otimes P_{\rm ex}(-\boldsymbol{f})$, where $P_{\rm ex}(\boldsymbol{f})$ is a pupil function of the excitation system. Interestingly, the frequency cutoff of the 3-D aperture does not change according to the detector size if $NA_{\rm ex} = NA_{\rm col}$. It is because $\widetilde{D}(\boldsymbol{f})$ is a sinc function (Fourier transform of rectangular function) or Bessel function (Fourier transform of cylindrical function) in the f_x-f_y directions and is uniform in the f_z direction.

Next, we consider Kohler illumination microscopy with a $\chi^{(1)}$-derived interaction, assuming $D(\boldsymbol{x}_{\rm d}) = \delta(\boldsymbol{x}_{\rm d})$. After multiplying $P_{\rm ill}(-\boldsymbol{f})$ of the excitation by the complex conjugate of $P_{\rm ill}(-\boldsymbol{f})P_{\rm col}(-\boldsymbol{f})$ of the LO and convolving it with $P_{\rm col}(\boldsymbol{f})$ of the signal, the 3-D aperture becomes $[|P_{\rm ill}(-\boldsymbol{f})|^2\{P_{\rm col}(-\boldsymbol{f})\}^*] \otimes P_{\rm col}(\boldsymbol{f})$. In contrast to laser microscopy that has a sufficiently large detector, meaning $\widetilde{D}(\boldsymbol{f}) = \delta(\boldsymbol{f})$, the functions of the illumination (excitation) and collection systems invert. Because the LO is present, the OTF can be defined as shown in Supplementary Figure 5 as an example of bright-field microscopy.

Supplementary Note 2: Category 2)

In category 2), a coherent system without the LO, we again begin with laser microscopy. We first consider the third harmonic generation, which is a $\chi^{(3)}$-derived interaction, as an example of the light-matter interaction, as shown in the diagram in Supplementary Figure 1. The 3-D aperture is found to be $A(\boldsymbol{f}) = P_{\rm ex}(-\boldsymbol{f}) \otimes P_{\rm ex}(-\boldsymbol{f}) \otimes P_{\rm ex}(-\boldsymbol{f}) \otimes \left\{P_{\rm col}(\boldsymbol{f})\widetilde{D_{\rm R}}(\boldsymbol{f})\right\}$ by convolving all the functions corresponding to each arrow. The frequency cutoff of the 3-D aperture does not vary based on the detector size; however, if the object observed is a single point, the Fourier transform of the image $\mathrm{AC}[\mathrm{A}(\boldsymbol{f})]$ changes according to the detector size. This results from the characteristic of the detector function: $\mathrm{AC}\left[\widetilde{D_{\rm R}}(\boldsymbol{f})\right] = \widetilde{D_{\rm R}}(\boldsymbol{f}) \otimes \left\{\widetilde{D_{\rm R}}(-\boldsymbol{f})\right\}^* = \widetilde{D}(\boldsymbol{f})$, where both $\widetilde{D_{\rm R}}(\boldsymbol{f})$ and $\widetilde{D}(\boldsymbol{f})$ are uniform in the f_z direction. In particular, when the detector size is sufficiently large, $\mathrm{AC}[\mathrm{A}(\boldsymbol{f})]$ becomes $\mathrm{AC}[P_{\rm ex}(-\boldsymbol{f}) \otimes P_{\rm ex}(-\boldsymbol{f}) \otimes P_{\rm ex}(-\boldsymbol{f})]$, while when it is sufficiently small, $A(\boldsymbol{f})$ is $\mathrm{AC}[P_{\rm ex}(-\boldsymbol{f}) \otimes P_{\rm ex}(-\boldsymbol{f}) \otimes P_{\rm ex}(-\boldsymbol{f}) \otimes P_{\rm col}(\boldsymbol{f})]$. This means that although the information on the object frequency inside the 3-D aperture reaches the detector, the image changes to some extent based on the detector size. A smaller detector size seemingly allows for a better optical resolution, forming a sharper image of a single point object, but the largest object frequency acquired is independent of the detector size. In addition, the OTF cannot be defined in this case without the LO.

Next, we consider Kohler illumination microscopy with a $\chi^{(1)}$-derived interaction under the condition of partially coherent image formation. In this case, the interference between the diffracted lights is not negligible and the LO is absent. The 3-D aperture can be calculated by convolving $P_{\mathrm{ill}}(-\boldsymbol{f})$ with $P_{\mathrm{col}}(\boldsymbol{f})$ in the relevant diagram (see Fig. 1 (b) ~ (d) of the main manuscript): $P_{\mathrm{ill}}(-\boldsymbol{f}) \otimes P_{\mathrm{col}}(\boldsymbol{f})$ (see also Eq. (11) of the main manuscript).

Supplementary Note 3: Category 3)

In category 3), an incoherent system with the LO, we take fluorescence as an example of the light-matter interaction. In fluorescence, which is one of the $\chi^{(3)}$-derived interactions, the vacuum field around the sample acts as the LO despite the fact that the vacuum field itself cannot be observed. First, we consider laser microscopy with a finite-sized single detector. The diagram (including the LO) is shown in Fig. 1 (b) of the main manuscript, where we define the 3-D pupil function of the vacuum field around the sample $V(\boldsymbol{f})$ as the dotted arrow, which has values in all directions with a random phase distribution (see the SI for more details). To obtain the 3-D aperture, we first treat $V(-\boldsymbol{f})$ of the vacuum as one of the excitations and multiply this by the complex conjugate of $\widetilde{D_R}(-\boldsymbol{f})V(-\boldsymbol{f})P_{\mathrm{col}}(-\boldsymbol{f})$ of the LO. We then convolve all of the functions corresponding to each arrow in the diagram to obtain $\mathrm{A}(\boldsymbol{f}) = P_{\mathrm{ex}}(-\boldsymbol{f}) \otimes P_{\mathrm{ex}}^{*}(\boldsymbol{f}) \otimes \left[\{P_{\mathrm{col}}^{*}(-\boldsymbol{f}) \otimes P_{\mathrm{col}}(\boldsymbol{f})\}\widetilde{D}(\boldsymbol{f})\right]$. Here, we use $|V(-\boldsymbol{f})|^2 P_{\mathrm{col}}(-\boldsymbol{f}) = P_{\mathrm{col}}(-\boldsymbol{f})$ and $\mathrm{AC}\left[\widetilde{D_{\mathrm{R}}}(\boldsymbol{f})\right] = \widetilde{D}(\boldsymbol{f})$. The OTF for the imaginary part of $\chi_{\mathrm{flu}}^{(3)}$ is defined as $OTF_{\mathrm{i}}(\boldsymbol{f}) = -A(\boldsymbol{f}) - A^{*}(-\boldsymbol{f})$, which is proportional to the 3-D aperture because $\mathrm{A}(\boldsymbol{f})$ is Hermitian in this case. This coincides with the Fourier transform of Eq. (19) in "Methods". In contrast, the OTF of the real part $OTF_{\mathrm{r}}(\boldsymbol{f}) = iA(\boldsymbol{f}) - iA^{*}(-\boldsymbol{f})$ inevitably vanishes in category 3).

Next, we consider Kohler illumination fluorescence microscopy. The 3-D aperture can be computed in the same manner. By replacing $P_{\mathrm{ex}}(\boldsymbol{f})$ with $P_{\mathrm{ill}}(\boldsymbol{f})$ and using the relation $P_{\mathrm{ill}}(-\boldsymbol{f}) \otimes P_{\mathrm{ill}}^{*}(\boldsymbol{f}) = \delta(\boldsymbol{f})$, the 3-D aperture reduces to $\mathrm{A}(\boldsymbol{f}) = P_{\mathrm{col}}^{*}(-\boldsymbol{f}) \otimes P_{\mathrm{col}}(\boldsymbol{f})$, assuming that the 2-D detector has a sufficiently small pixel size. The OTF of the imaginary part is also proportional to the 3-D aperture in this case, which is consistent with the well-known OTF of wide-field fluorescence microscopy[1].

Supplementary Note 4: Category 4)

In category 4), an incoherent system without the LO, we use spontaneous parametric down-conversion (SPDC) as an example of the light-matter interaction. In SPDC, two fields referred to as the signal and idler are generated by the excitation filed[2]. The diagram of SPDC, which is a $\chi^{(2)}$-derived interaction, is described in Fig. 1 (b) of the main manuscript, where we presume that only the signal is observed; the idler is not detected. Again, we start with laser microscopy. By

convolving all the functions corresponding to each arrow, the 3-D aperture is found to be $P_{\mathrm{ex}}(-\boldsymbol{f}) \otimes V^*(\boldsymbol{f}) \otimes \{\widetilde{D_{\mathrm{R}}}(\boldsymbol{f}) P_{\mathrm{col}}(\boldsymbol{f})\}$, where $V^*(\boldsymbol{f})$ for this case corresponds to the idler field. The frequency cutoff of the 3-D aperture does not vary by detector size, while the Fourier transform of the image of a single point object $\tilde{S}(\boldsymbol{f})$ does change according to the detector size: $\tilde{S}(\boldsymbol{f}) = \mathrm{AC}[P_{\mathrm{ex}}(-\boldsymbol{f})] \otimes \{\mathrm{AC}[P_{\mathrm{col}}(\boldsymbol{f})] \widetilde{D}(\boldsymbol{f})\}$, thus, the relation $\mathrm{AC}[V^*(\boldsymbol{f})] = \delta(\boldsymbol{f})$ is used. This is also one of our findings.

Next, we consider Kohler illumination microscopy in the context of SPDC. The 3-D aperture can be calculated in the same manner. By replacing $P_{\mathrm{ex}}(\boldsymbol{f})$ with $P_{\mathrm{ill}}(\boldsymbol{f})$, the 3-D aperture becomes $P_{\mathrm{ill}}(-\boldsymbol{f}) \otimes V^*(\boldsymbol{f}) \otimes P_{\mathrm{col}}(\boldsymbol{f})$, assuming that the 2-D detector has a sufficiently small pixel size. Note that in the case of coincidence photon counting of the signal and idler, the dotted arrow of the idler in the diagram changes to a wavy arrow. In this case, two wavy arrows corresponding to the signal and idler exist in a diagram, as shown in Supplementary Figure 1, which means that the coherence revives despite the incoherent light-matter interaction involved in the vacuum field.

Supplementary Note 5: Theorem of resolution limit

We consider optical microscopy to be composed of photoexcitation and photodetection systems, where all of the light-matter interactions occurring in the sample are addressed. For the excitation system, two principal types are employed, i.e., the Kohler illumination system with the incoherent surface light source and the laser excitation system. Because the pupil function corresponding to the Kohler illumination system possesses a random phase distribution, the 3-D aperture in this case does not surpass that of laser-scanning microscopy. In the detection system, the detector size, such as the size of a pinhole or the pixel size of a two-dimensional detector, affects the 3-D aperture. The frequency cutoff of the 3-D aperture is largest when the pixel size or the pinhole size is sufficiently small. Therefore, confocal laser microscopy is one of the systems that has the largest frequency cutoff of the 3-D aperture. In confocal laser microscopy, the 3-D aperture is determined by the NAs of the excitation and detection systems, the wavelengths of the excitation beams and detection light, and the type of light-matter interactions.

The Feynman diagram expresses light-matter interactions, including information on the NAs and the wavelengths in terms of pupil functions. Thus, we propose a theorem concerning the resolution limit of a microscope system: for a given type of light-matter interaction, the Feynman diagram determines the upper limit of the 3-D aperture for far-field optical microscopy if *a priori* information on the sample does not exist. Even in the case of an enhanced high signal-to-noise ratio or a different type of excitation/signal-collection system from laser-scanning and Kohler illumination microscopy, the object frequency outside the upper limit determined by the Feynman diagram is never acquired. This holds true even for structured illumination microscopy[3], image

scanning microscopy[4], and stimulated emission depletion microscopy[5].

Supplementary Note 6: Real space representation

In order to address optical microscopy, we move from the frequency space to the real space. The plane wave of an excitation laser beam can be assumed to be in a coherent state with a frequency of $\boldsymbol{f} = (f_x, f_y, f_z)$:

$$|\alpha\rangle_{\boldsymbol{f}} = e^{-\frac{|\alpha|^2}{2}} \sum_{n=0}^{\infty} \frac{\alpha^n}{\sqrt{n!}} |n\rangle_{\boldsymbol{f}}, \tag{1}$$

where α is a complex number and $|n\rangle_{\boldsymbol{f}}$ is the number state for the plane wave with frequency $\boldsymbol{f}$ (wavenumber $\boldsymbol{k}$=2π$\boldsymbol{f}$)[6]. Because the laser beam is focused on the sample by the excitation objective, the corresponding excitation state is represented by the direct product of all modes restricted by the NA and wavelength:

$$|\alpha\rangle_{\mathrm{ex}} = \prod_{\boldsymbol{f} \in P_{\mathrm{ex}}(\boldsymbol{f})} |\alpha\rangle_{\boldsymbol{f}}, \tag{2}$$

where $P_{\mathrm{ex}}(\boldsymbol{f})$ represents the 3-D pupil function for the excitation objective. In coherent light-matter interactions, we consider only the coherent states for laser beams. When two different laser beams (ex1 and ex2) are employed for the excitation, the state representing the excitation condition becomes $|\alpha\rangle_{\mathrm{ex1}}|\alpha\rangle_{\mathrm{ex2}}$.

Supplementary Note 7: The vacuum state in microscopy

For incoherent light-matter interactions, we incorporate the vacuum state $|0\rangle$, which exists around the sample, into the formulation as one of the excitation sources. For this purpose, we consider the direct product of the coherent state and vacuum state $|\alpha\rangle_{\mathrm{ex}}|0\rangle$ as the excitation condition. The vacuum state $|0\rangle$ contains all modes $|0\rangle_{\boldsymbol{f}}$ with frequencies $\boldsymbol{f}$:

$$|0\rangle = \prod_{\boldsymbol{f}} |0\rangle_{\boldsymbol{f}}. \tag{3}$$

Supplementary Note 8: Operator in real space for microscopy

We now introduce the basic idea of the annihilation and creation operators in real space, $\hat{a}(\boldsymbol{x})$ and $\hat{a}^{+}(\boldsymbol{x})$, using the 3-D pupil function $P(\boldsymbol{f})$:

$$\hat{a}(\boldsymbol{x}) = \int P(\boldsymbol{f})\, \hat{a}(\boldsymbol{f}) e^{i2\pi \boldsymbol{f}\cdot \boldsymbol{x}} d^3\boldsymbol{f}, \tag{4}$$

$$\hat{a}^{+}(\boldsymbol{x}) = \int P^{*}(\boldsymbol{f})\, \hat{a}^{+}(\boldsymbol{f}) e^{-i2\pi \boldsymbol{f}\cdot \boldsymbol{x}} d^3\boldsymbol{f}, \tag{5}$$

where $\hat{a}(\boldsymbol{f})$ and $\hat{a}^{+}(\boldsymbol{f})$ are the annihilation and creation operators in frequency space, respectively. In a

similar way, we can define the annihilation operators in real space for the excitation laser field $\hat{a}_{\mathrm{ex}}(\boldsymbol{x})$, the vacuum field around the sample $\hat{a}_{\mathrm{vac}}(\boldsymbol{x})$, the local oscillator field derived from the vacuum field $\hat{a}_{\mathrm{lo(v)}}(\boldsymbol{x})$, the local oscillator field due to the excitation laser field $\hat{a}_{\mathrm{lo(l)}}(\boldsymbol{x})$, the signal field emitted from the sample $\hat{a}_{\mathrm{sig}}(\boldsymbol{x})$, and the signal field collected by the detector $\hat{a}_{\mathrm{col}}(\boldsymbol{x})$ as:

$$\hat{a}_{\mathrm{ex}}(\boldsymbol{x}) = \int P_{\mathrm{ex}}(\boldsymbol{f})\,\hat{a}(\boldsymbol{f})e^{i2\pi\boldsymbol{f}\cdot\boldsymbol{x}}d^3\boldsymbol{f}, \tag{6}$$

$$\hat{a}_{\mathrm{vac}}(\boldsymbol{x}) = \int V(\boldsymbol{f})\delta\big(|\boldsymbol{f}| - f_{\mathrm{sig}}\big)\,\hat{a}(\boldsymbol{f})e^{i2\pi\boldsymbol{f}\cdot\boldsymbol{x}}d^3\boldsymbol{f}, \tag{7}$$

$$\hat{a}_{\mathrm{lo(v)}}(\boldsymbol{x}_{\mathrm{d}}) = \int V(\boldsymbol{f}_{\mathrm{d}})\,P_{\mathrm{col}}(\boldsymbol{f}_{\mathrm{d}})\hat{a}(\boldsymbol{f}_{\mathrm{d}})e^{i2\pi\boldsymbol{f}_{\mathrm{d}}\cdot\boldsymbol{x}_{\mathrm{d}}}d^3\boldsymbol{f}_{\mathrm{d}}, \tag{8}$$

$$\hat{a}_{\mathrm{lo(l)}}(\boldsymbol{x}_{\mathrm{d}}) = \int P_{\mathrm{ex}}(\boldsymbol{f}_{\mathrm{d}})\,P_{\mathrm{col}}(\boldsymbol{f}_{\mathrm{d}})\hat{a}(\boldsymbol{f}_{\mathrm{d}})e^{i2\pi\boldsymbol{f}_{\mathrm{d}}\cdot\boldsymbol{x}_{\mathrm{d}}}d^3\boldsymbol{f}_{\mathrm{d}}, \tag{9}$$

$$\hat{a}_{\mathrm{sig}}(\boldsymbol{x}) = \int \delta_{+}(|\boldsymbol{f}| - f_{\mathrm{sig}})\,\hat{a}(\boldsymbol{f})e^{i2\pi\boldsymbol{f}\cdot\boldsymbol{x}}d^3\boldsymbol{f}, \tag{10}$$

$$\hat{a}_{\mathrm{col}}(\boldsymbol{x}_{\mathrm{d}}) = \int P_{\mathrm{col}}(\boldsymbol{f}_{\mathrm{d}})\hat{a}(\boldsymbol{f}_{\mathrm{d}})e^{i2\pi\boldsymbol{f}_{\mathrm{d}}\cdot\boldsymbol{x}_{\mathrm{d}}}\,d^3\boldsymbol{f}_{\mathrm{d}}, \tag{11}$$

$$\hat{a}_{\mathrm{ill}}(\boldsymbol{x}) = \int P_{\mathrm{ill}}(\boldsymbol{f})\,\hat{a}(\boldsymbol{f})e^{i2\pi\boldsymbol{f}\cdot\boldsymbol{x}}d^3\boldsymbol{f}, \tag{12}$$

$$\hat{a}_{\mathrm{det}}(\boldsymbol{x}_{\mathrm{d}}) = \int \hat{a}(\boldsymbol{f}_{\mathrm{d}})\,e^{i2\pi\boldsymbol{f}_{\mathrm{d}}\cdot\boldsymbol{x}_{\mathrm{d}}}d^3\boldsymbol{f}_{\mathrm{d}} \tag{13}$$

with

$$P_{\mathrm{ex}}(f_x, f_y, f_z) = P_{\mathrm{ex}}^{(2)}(f_x, f_y)P_{\mathrm{ex}}^{(3)}(f_x, f_y, f_z), \tag{14}$$

$$\delta_{+}\big(|\boldsymbol{f}| - f_{\mathrm{sig}}\big) = \delta\big(|\boldsymbol{f}| - f_{\mathrm{sig}}\big) + \frac{1}{i\pi\big(|\boldsymbol{f}| - f_{\mathrm{sig}}\big)}. \tag{15}$$

Here, $P_{\mathrm{ex}}(\boldsymbol{f})$ is the 3-D pupil function for the excitation system expressed by the product of the 2-D pupil function for the excitation system $P_{\mathrm{ex}}^{(2)}(f_x, f_y)$ (which includes the laser beam profile) and the spherical shell truncated by *NA* $P_{\mathrm{ex}}^{(3)}(f_x, f_y, f_z)$, which has delta-function characteristics in the radial direction. $V(\boldsymbol{f})$, describing the vacuum field, represents a complex random function whose modulus is unity, $P_{\mathrm{col}}(\boldsymbol{f})$ is the 3-D pupil function for the signal-collection system, which is the partial sphere with a modulus of one, and f_{sig} is the modulus of the wavenumber for the signal field. Note that f_{sig} considers the refractive index of the sample. Information about the aberration is included in the pupil functions. Thus, we define the operators describing the excitation and signal fields in microscopy, which also allows for the calculation associated with the vacuum field.

Supplementary Note 9: Conversion of q-number into c-number

We convert q-number (operator) to c-number by acting on the state. To unify the framework for

coherent and incoherent light-matter interactions, the classical field is replaced by the operator. Then, the operator acts on the bra or ket describing the excitation condition. Because the vacuum field inevitably exists around the sample, we always include both the coherent state for the laser and the vacuum state in the excitation condition, i.e. as $|\alpha\rangle_{\mathrm{ex}}|0\rangle$. For example, using the relation $\hat{a}_{\mathrm{ex}}(\boldsymbol{f})|\alpha\rangle_{\boldsymbol{f}} = \alpha|\alpha\rangle_{\boldsymbol{f}}$, the calculation is as follows:

$$\hat{a}_{\mathrm{ex}}(\boldsymbol{x})|\alpha\rangle_{\mathrm{ex}} = \int P_{\mathrm{ex}}(\boldsymbol{f})\,\hat{a}(\boldsymbol{f})e^{i2\pi\boldsymbol{f}\cdot\boldsymbol{x}}d^3\boldsymbol{f}\;|\alpha\rangle_{\mathrm{ex}}$$
$$= \alpha ASF_{\mathrm{ex}}(\boldsymbol{x})\;|\alpha\rangle_{\mathrm{ex}}, \quad (16)$$

where $ASF_{\mathrm{ex}}(\boldsymbol{x})$ is the amplitude spread function (ASF) formed by the excitation laser beam on the sample through the excitation objective. The calculation related to the vacuum field is as follows:

$$\langle 0|\hat{a}_{\mathrm{col}}(\boldsymbol{x}_{\mathrm{d}})\hat{a}^{+}_{\mathrm{sig}}(\boldsymbol{x})|0\rangle = \int P_{\mathrm{col}}(\boldsymbol{f})\delta^{*}_{+}(|\boldsymbol{f}| - f_{\mathrm{sig}})\,e^{i2\pi\boldsymbol{f}\cdot(\boldsymbol{x}_{\mathrm{d}}-\boldsymbol{x})}d^3\boldsymbol{f}$$
$$= h_{\mathrm{col}}(\boldsymbol{x}_{\mathrm{d}} - \boldsymbol{x}), \quad (17)$$
$$\langle 0|\hat{a}_{\mathrm{vac}}(\boldsymbol{x})\hat{a}^{+}_{\mathrm{lo(v)}}(\boldsymbol{x}_{\mathrm{d}})|0\rangle = \left\{\int |V(\boldsymbol{f})|^2 P_{\mathrm{col}}(\boldsymbol{f})\delta(|\boldsymbol{f}| - f_{\mathrm{sig}})\,e^{i2\pi\boldsymbol{f}\cdot(\boldsymbol{x}_{\mathrm{d}}-\boldsymbol{x})}d^3\boldsymbol{f}\right\}^{*}$$
$$= \{h_{\mathrm{col}}(\boldsymbol{x}_{\mathrm{d}} - \boldsymbol{x})\}^{*}, \quad (18)$$
$$\langle 0|\hat{a}_{\mathrm{sig}}(\boldsymbol{x})\hat{a}^{+}_{\mathrm{col}}(\boldsymbol{x}_{\mathrm{d}})|0\rangle = \left\{\langle 0|\hat{a}_{\mathrm{col}}(\boldsymbol{x}_{\mathrm{d}})\hat{a}^{+}_{\mathrm{sig}}(\boldsymbol{x})|0\rangle\right\}^{*}$$
$$= \{h_{\mathrm{col}}(\boldsymbol{x}_{\mathrm{d}} - \boldsymbol{x})\}^{*}, \quad (19)$$

where $h_{\mathrm{col}}(\boldsymbol{x}_{\mathrm{d}})$ is the ASF formed by the signal field on the detector through the signal-collection objective. Supplementary Equation (17) represents the propagator for the photon created at $\boldsymbol{x}$ and annihilated at $\boldsymbol{x}_{\mathrm{d}}$. In Supplementary Equations (18) and (19), the left-hand side, which appears to be the light propagation from $\boldsymbol{x}_{\mathrm{d}}$ to $\boldsymbol{x}$, physically indicates that the light expressed by the complex conjugate propagates from $\boldsymbol{x}$ to $\boldsymbol{x}_{\mathrm{d}}$. Note that in the table 1 in "Results", we rewrite $P_{\mathrm{col}}(\boldsymbol{f})\delta^{*}_{+}(|\boldsymbol{f}| - \boldsymbol{f}_{\mathrm{sig}})$ and $V(\boldsymbol{f})\delta(|\boldsymbol{f}| - f_{\mathrm{sig}})$ as $P_{\mathrm{col}}(\boldsymbol{f})$ and $V(\boldsymbol{f})$, respectively, which have delta-function characteristics in the radial direction.

Supplementary Note 10: Commutation relation of operators

For the convenience of formula transformation and simplification, we calculate the commutation relation between the annihilation and creation operators in real space:

$$[\hat{a}_{\mathrm{col}}(\boldsymbol{x}_{\mathrm{d}}), \hat{a}^{+}_{\mathrm{col}}(\boldsymbol{x}_{\mathrm{d}})]$$
$$= \hat{a}_{\mathrm{col}}(\boldsymbol{x}_{\mathrm{d}})\,\hat{a}^{+}_{\mathrm{col}}(\boldsymbol{x}_{\mathrm{d}}) - \hat{a}^{+}_{\mathrm{col}}(\boldsymbol{x}_{\mathrm{d}})\hat{a}_{\mathrm{col}}(\boldsymbol{x}_{\mathrm{d}})$$
$$= \int P_{\mathrm{col}}(\boldsymbol{f}_1)\hat{a}(\boldsymbol{f}_1)e^{i2\pi\boldsymbol{f}_1\cdot\boldsymbol{x}_{\mathrm{d}}}\,d^3\boldsymbol{f}_1 \int P^{*}_{\mathrm{col}}(\boldsymbol{f}_2)\hat{a}^{+}(\boldsymbol{f}_2)e^{-i2\pi\boldsymbol{f}_2\cdot\boldsymbol{x}_{\mathrm{d}}}\,d^3\boldsymbol{f}_2$$
$$- \int P^{*}_{\mathrm{col}}(\boldsymbol{f}_2)\hat{a}^{+}(\boldsymbol{f}_2)e^{-i2\pi\boldsymbol{f}_2\cdot\boldsymbol{x}_{\mathrm{d}}}\,d^3\boldsymbol{f}_2 \int P_{\mathrm{col}}(\boldsymbol{f}_1)\hat{a}(\boldsymbol{f}_1)e^{i2\pi\boldsymbol{f}_1\cdot\boldsymbol{x}_{\mathrm{d}}}\,d^3\boldsymbol{f}_1$$
$$= \iint P_{\mathrm{col}}(\boldsymbol{f}_1)\,P^{*}_{\mathrm{col}}(\boldsymbol{f}_2)\{\hat{a}(\boldsymbol{f}_1)\hat{a}^{+}(\boldsymbol{f}_2) - \hat{a}^{+}(\boldsymbol{f}_2)\hat{a}(\boldsymbol{f}_1)\}e^{i2\pi(\boldsymbol{f}_1-\boldsymbol{f}_2)\cdot\boldsymbol{x}_{\mathrm{d}}}d^3\boldsymbol{f}_1 d^3\boldsymbol{f}_2$$
$$= \iint P_{\mathrm{col}}(\boldsymbol{f}_1)\,P^{*}_{\mathrm{col}}(\boldsymbol{f}_2)[\hat{a}(\boldsymbol{f}_1), \hat{a}^{+}(\boldsymbol{f}_2)]e^{i2\pi(\boldsymbol{f}_1-\boldsymbol{f}_2)\cdot\boldsymbol{x}_{\mathrm{d}}}d^3\boldsymbol{f}_1 d^3\boldsymbol{f}_2$$
$$= \iint P_{\mathrm{col}}(\boldsymbol{f}_1)\,P^{*}_{\mathrm{col}}(\boldsymbol{f}_2)\delta(\boldsymbol{f}_1 - \boldsymbol{f}_2)e^{i2\pi(\boldsymbol{f}_1-\boldsymbol{f}_2)\cdot\boldsymbol{x}_{\mathrm{d}}}d^3\boldsymbol{f}_1 d^3\boldsymbol{f}_2$$

$$= \int |P_{\mathrm{col}}(\boldsymbol{f})|^2 \, d^3\boldsymbol{f}, \tag{20}$$

using the commutation relation in the frequency domain $[\hat{a}(\boldsymbol{f}_1), \hat{a}^{+}(\boldsymbol{f}_2)] = \delta(\boldsymbol{f}_1 - \boldsymbol{f}_2)$[6]. Likewise, we obtain

$$\begin{aligned}\left[\hat{a}_{\mathrm{col}}(\boldsymbol{x}_{\mathrm{d}}), \hat{a}^{+}_{\mathrm{lo(v)}}(\boldsymbol{x}_{\mathrm{d}})\right] &= \int V^{*}(\boldsymbol{f}_{\mathrm{d}})|P_{\mathrm{col}}(\boldsymbol{f}_{\mathrm{d}})|^2 d^3\boldsymbol{f}_{\mathrm{d}} \\ &= 0, \end{aligned} \tag{21}$$

using the random phase nature of $V^{*}(\boldsymbol{f}_{\mathrm{d}})$. Thus, we formulate the commutation relation of operators in real space by using the conventional operators in frequency space to address microscope system.

Supplementary Figures

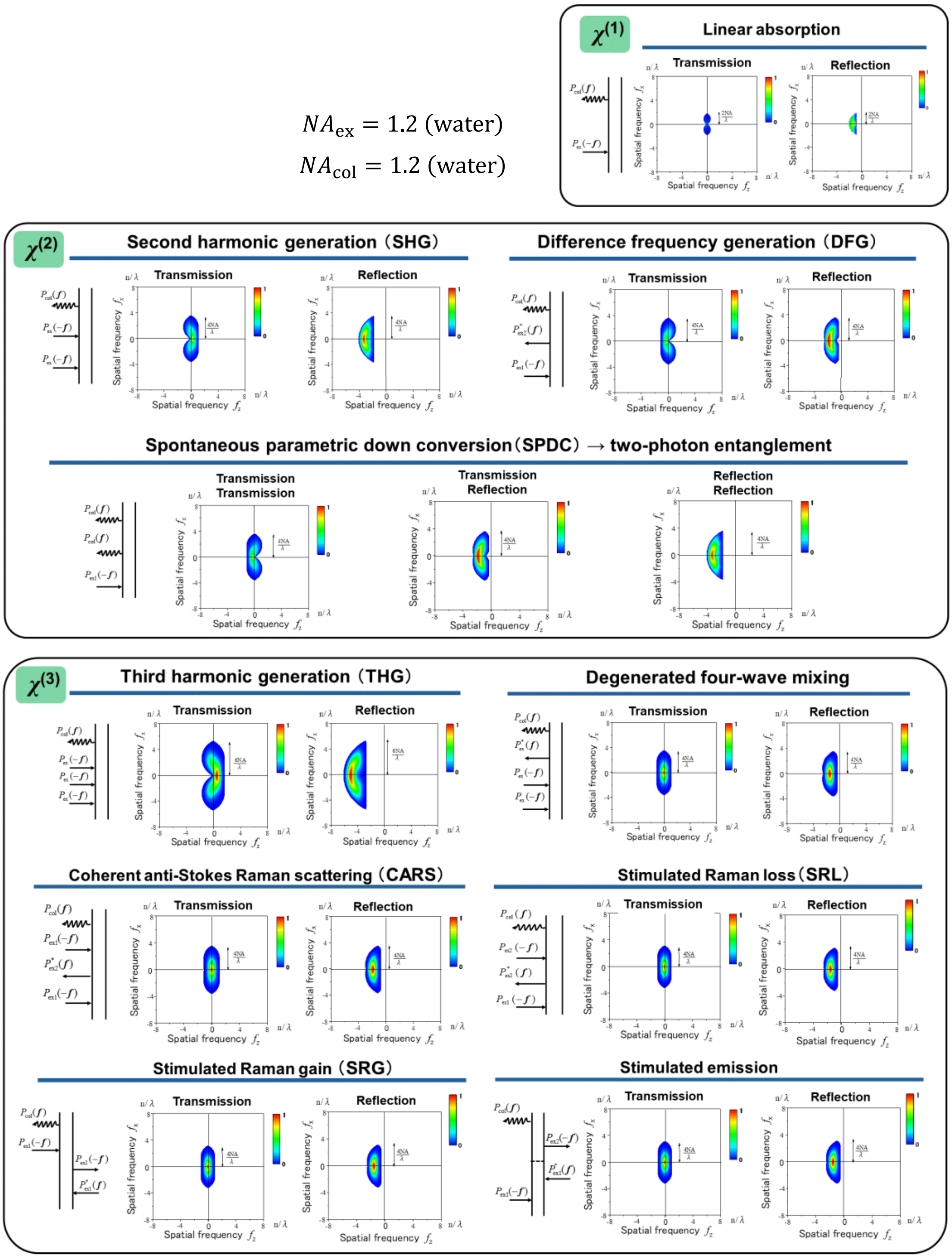

Supplementary Figure 1. 3-D apertures of confocal microscopy (transmission and reflection types) with various light-matter interactions categorized by $\chi^{(i)}$ (i=1~3). Feynman diagram corresponding

to each interaction is shown to the left of the 3-D apertures. 3-D aperture is calculated by convolving all 3-D pupil functions in the diagram. All panels show the f_x-f_z cross-sections, and they are rotationally symmetric around f_z axis. The scale bars to the right of each panel indicate the frequency cutoff in f_x-f_y directions as a guide. *n* is the average refractive index in the sample. λ is the wavelength of the excitation beam or the average wavelength if two excitation beams are used. The numerical apertures of the excitation and signal-correction systems are $NA_{\mathrm{ex}} = NA_{\mathrm{col}} = 1.2$.

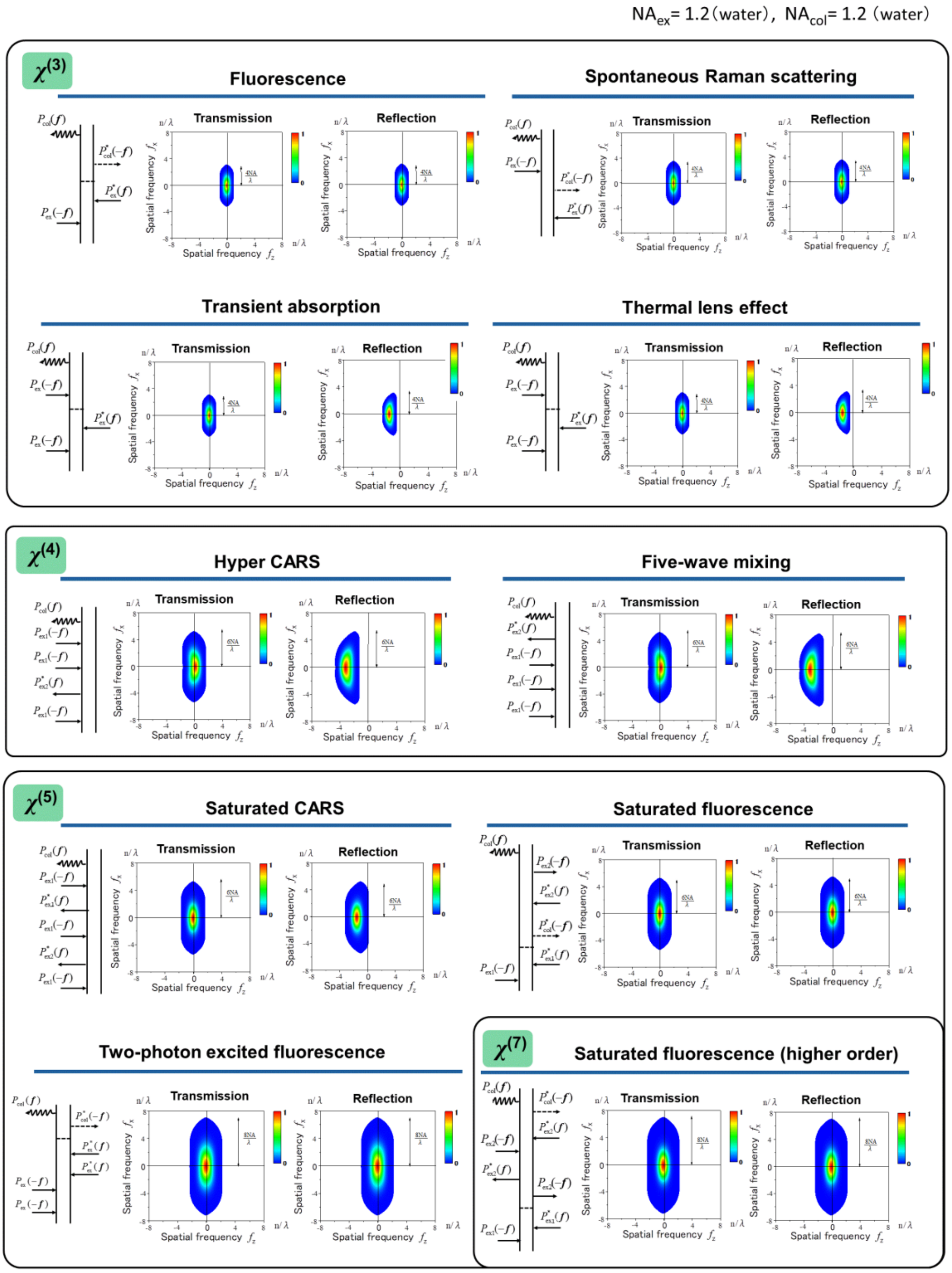

Supplementary Figure 2. 3-D apertures of confocal microscopy (transmission and reflection types) with various light-matter interactions categorized by $\chi^{(i)}$ (i=3~7). Feynman diagram corresponding to each interaction is shown to the left of the 3-D apertures. 3-D aperture is calculated by convolving

all 3-D pupil functions in the diagram. All panels show the f_x-f_z cross-sections, and they are rotationally symmetric around f_z axis. The scale bars to the right of each panel indicate the frequency cutoff in f_x-f_y directions as a guide. In incoherent interactions, the vacuum field (the dotted arrow) is one of the excitation fields, where we assume that Stokes shift is sufficiently small. n is the average refractive index in the sample. λ is the wavelength of the excitation beam or the average wavelength if two excitation beams are used. The numerical apertures of the excitation and signal-correction systems are $NA_{\mathrm{ex}} = NA_{\mathrm{col}} = 1.2$.

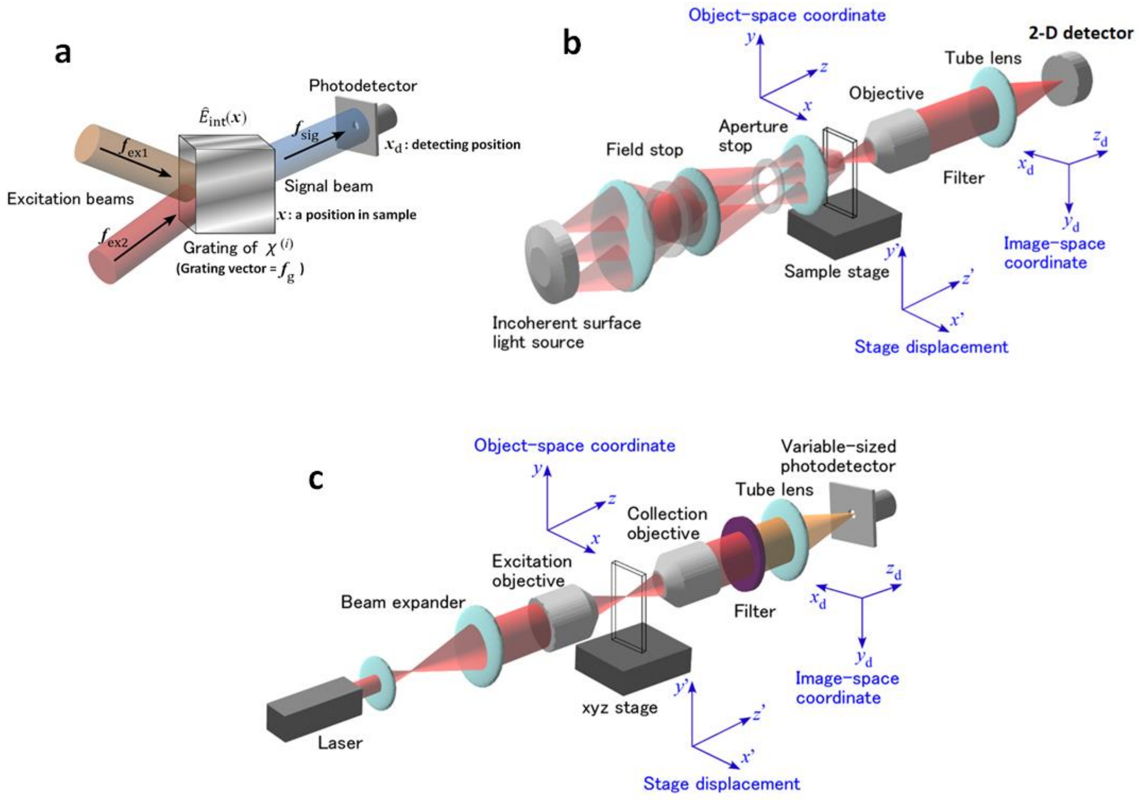

Supplementary Figure 3. Generalization of diffraction and typical optical microscope systems. Some microscopy employ a higher-order light-matter interaction than the $\chi^{(1)}$-derived interaction such as transmission. In this case, the concept of diffraction has to be generalized to quasi-phase-matching. (**a**) Illustration quasi-phase-matching representing sum frequency generation with $\chi^{(2)}$ grating; (**b**) Schematic of Kohler illumination microscopy; (**c**) Schematic of laser microscopy.

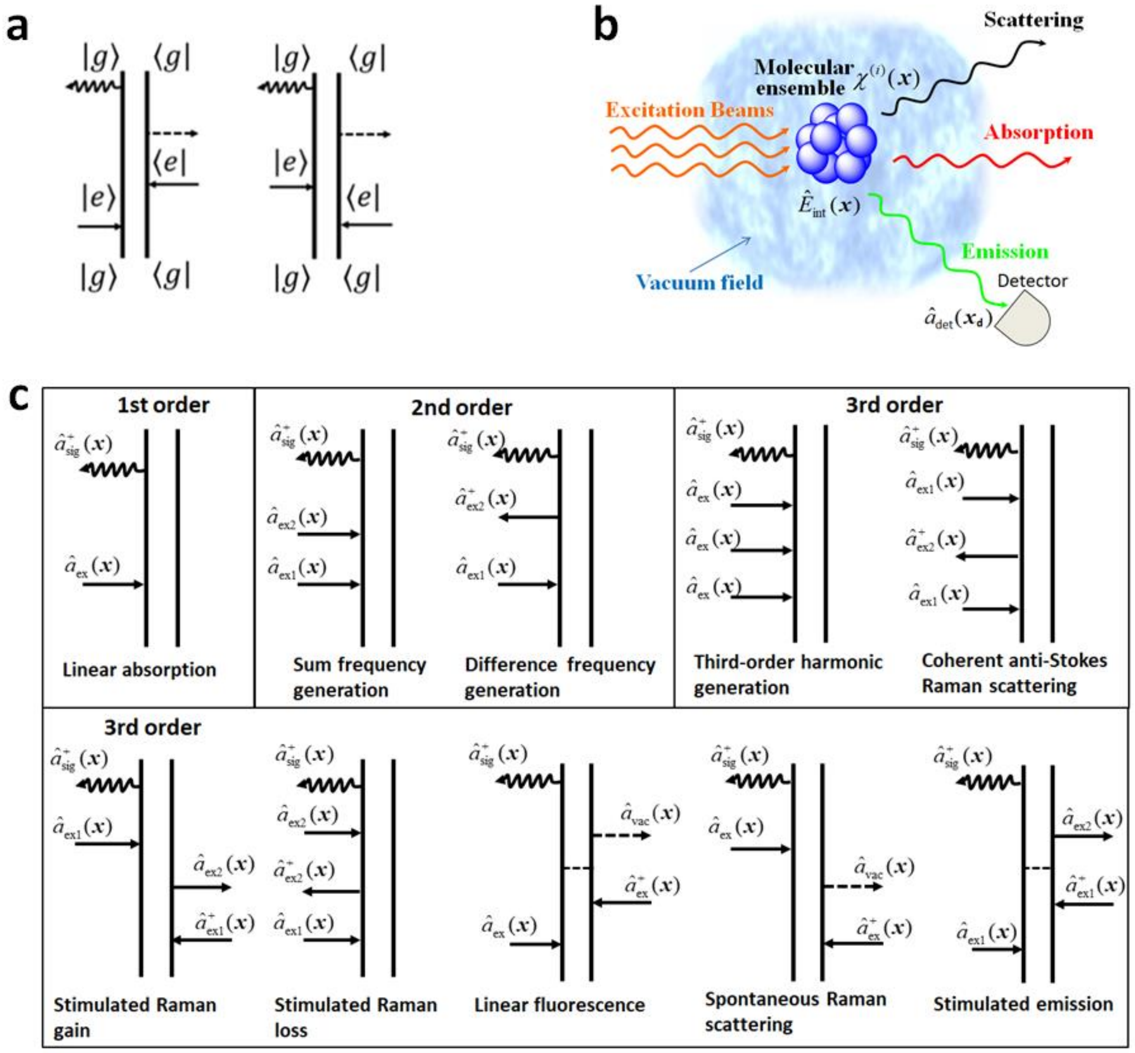

Supplementary Figure 4. Light-matter interactions. (**a**) Feynman diagrams describing fluorescence. The solid, dotted, and wavy arrows represent the excitation, vacuum, and signal fields, respectively. For fluorescence, two diagrams exist that differ in the order of the electric field that first excites the ket side $|g\rangle$ or bra side $\langle g|$. $|g\rangle$ and $|e\rangle$ represent the ground and excited states, respectively. Sequential excitation by two electric fields $\leftarrow (\hat{a}^{+}_{\text{ex}})$ and $\rightarrow (\hat{a}_{\text{ex}})$ correspond to a photon. (**b**) Interactions between molecular ensemble and light. The vacuum field is involved in incoherent interactions. (**c**) Examples of Feynman diagrams describing interactions such as sum frequency generation, third-order harmonic generation, coherent anti-Stokes Raman scattering, and spontaneous Raman scattering. Only one diagram is shown to represent an interaction, however in reality multiple simultaneous diagrams exist.

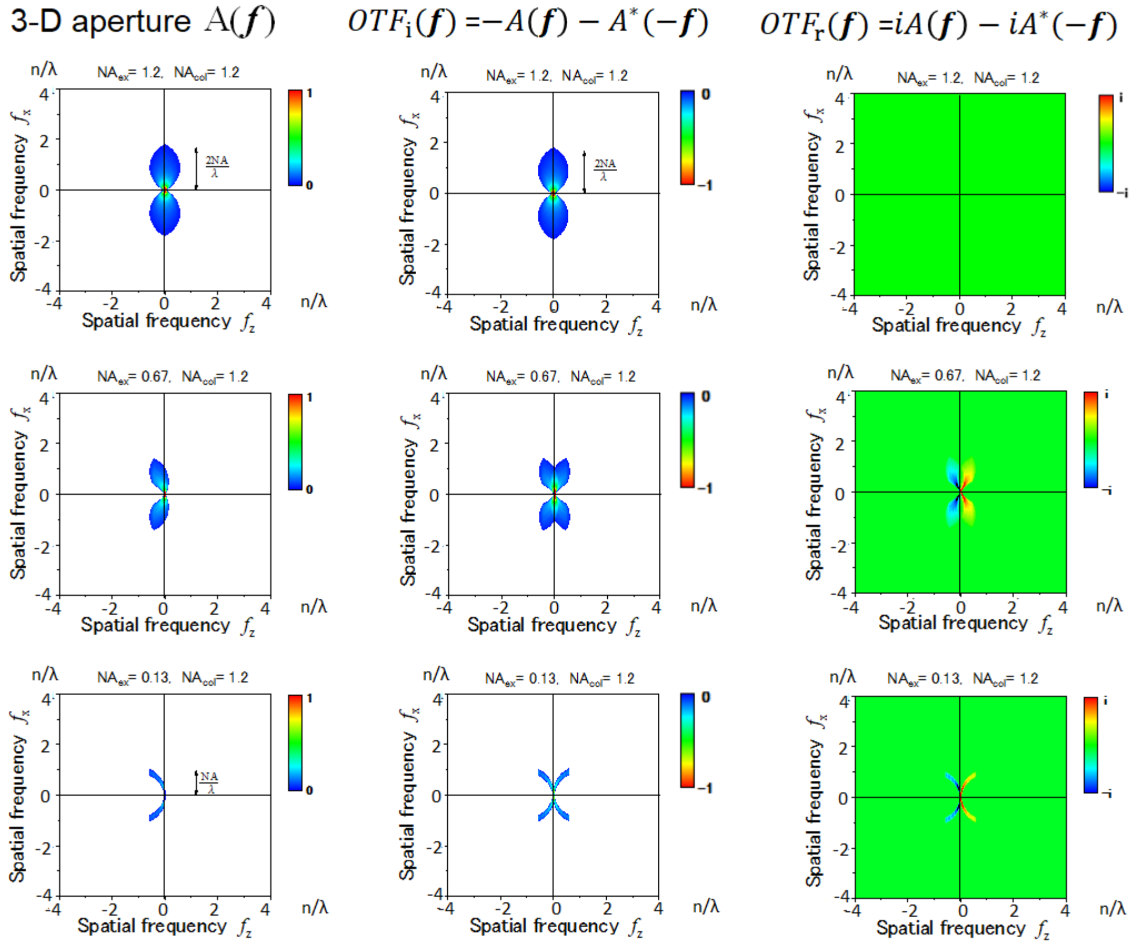

Supplementary Figure 5. 3-D aperture and two types of optical transfer functions (OTFs) of bright-field microscopy. All panels show the f_x-f_z cross-sections, and they are rotationally symmetric around f_z axis. $\mathrm{OTF_i}$ and $\mathrm{OTF_r}$ express the optical resolutions for $\mathrm{Im}\{\chi^{(1)}(\boldsymbol{x})\}$ corresponding to the absorption-rate distribution and $\mathrm{Re}\{\chi^{(1)}(\boldsymbol{x})\}$ corresponding to the refractive index distribution, respectively. The scale bars to the right of each panel indicate the frequency cutoff in f_x-f_y directions. n is the average refractive index in the sample, and λ is the wavelength of the illumination light. The numerical aperture of signal-correction system $NA_{\mathrm{col}} = 1.2$ (water) is fixed, and that of illumination system NA_{ex} varies: NA_{ex}=1.2 (top), 0.67 (middle), and 0.13 (bottom).

Supplementary References

1. Goodman, J. W. *Introduction to Fourier Optics*. *3rd ed.* (Roberts and Company Publishers, Greenwood Village, 2005).
2. Pittman, T. B., Shih, Y. H., Strekalov, D. V. & Sergienko, A. V. Optical imaging by means of two-photon quantum entanglement. *Phys. Rev. A*, **52**, R3429 (1995).
3. Gustafsson, M. G. Surpassing the lateral resolution limit by a factor of two using structured illumination microscopy. *J. Microsc.* **198**, 82-87 (2000).
4. Muller, C. B. & Enderlein, J. Image scanning microscopy. *Phys. Rev. Lett.* **104**, 198101 (2010).
5. Hell, S. W. & Wichmann, J. Breaking the diffraction resolution limit by stimulated emission: stimulated-emission-depletion fluorescence microscopy. *Opt. Lett.* **19**, 780–782 (1994).
6. Loudon, R. *The Quantum Theory of Light*. *3rd ed.* (Oxford Science Publications, Oxford, 2000).
